# The heparan sulfate proteoglycan agrin contributes to barrier properties of mouse brain endothelial cells by stabilizing adherens junctions

**DOI:** 10.1007/s00441-014-1969-7

**Published:** 2014-08-09

**Authors:** Esther Steiner, Gaby U. Enzmann, Ruth Lyck, Shuo Lin, Markus A. Rüegg, Stephan Kröger, Britta Engelhardt

**Affiliations:** 1 Theodor Kocher Institute, University of Bern, Freiestrasse 1, 3012 Bern, Switzerland; 2 Biocenter, University of Basel, 4056 Basel, Switzerland; 3 Institute for Physiology, Ludwig Maximillian University, 80336 Munich, Germany

**Keywords:** Blood–brain barrier, Agrin, Basement membrane, Adherens junctions, Tight junctions

## Abstract

Barrier characteristics of brain endothelial cells forming the blood–brain barrier (BBB) are tightly regulated by cellular and acellular components of the neurovascular unit. During embryogenesis, the accumulation of the heparan sulfate proteoglycan agrin in the basement membranes ensheathing brain vessels correlates with BBB maturation. In contrast, loss of agrin deposition in the vasculature of brain tumors is accompanied by the loss of endothelial junctional proteins. We therefore wondered whether agrin had a direct effect on the barrier characteristics of brain endothelial cells. Agrin increased junctional localization of vascular endothelial (VE)-cadherin, β-catenin, and zonula occludens-1 (ZO-1) but not of claudin-5 and occludin in the brain endothelioma cell line bEnd5 without affecting the expression levels of these proteins. This was accompanied by an agrin-induced reduction of the paracellular permeability of bEnd5 monolayers. In vivo, the lack of agrin also led to reduced junctional localization of VE-cadherin in brain microvascular endothelial cells. Taken together, our data support the notion that agrin contributes to barrier characteristics of brain endothelium by stabilizing the adherens junction proteins VE-cadherin and β-catenin and the junctional protein ZO-1 to brain endothelial junctions.

**Electronic supplementary material:**

The online version of this article (doi:10.1007/s00441-014-1969-7) contains supplementary material, which is available to authorized users.

## Introduction

The blood–brain barrier (BBB) is established at the level of brain microvessels by highly specialized endothelial cells that inhibit the free paracellular diffusion of water soluble molecules by an elaborate network of complex tight junctions (TJs). Combined with the absence of fenestrae and an extremely low pinocytotic activity, which inhibit the transcellular passage of molecules across the barrier, these morphological features establish the physical permeability barrier of the BBB (Engelhardt and Sorokin 2009). In addition, a number of permanently active transport mechanisms expressed at the level of brain microvascular endothelial cells ensure the transport of nutrients into the central nervous system (CNS) and exclusion of toxic metabolites out of the CNS. Thus, the BBB maintains brain homeostasis, a prerequisite for proper communication between brain neurons. Moreover, whereas the endothelial cells constitute the physical and metabolic barrier per se, barrier characteristics are not an intrinsic feature of these endothelial cells, but rather rely on the interactions with adjacent cellular and acellular layers of brain microvessels (Engelhardt and Sorokin 2009). The fully differentiated BBB consists of the highly specialized endothelial cells and their underlying endothelial basement membrane, in which a large number of pericytes are embedded. These microvessels are surrounded by an ensheathment of astrocytic endfeet and their associated parenchymal basement membrane referred to as the glia limitans. As it is now well established, both pericytes and astrocytes actively contribute, with their soluble mediators, to the development and maintenance of barrier characteristics in brain microvascular endothelial cells (Engelhardt and Sorokin 2009). In contrast, the relevance of acellular components, such as the extracellular matrix (ECM) proteins collagen type IV, various laminin isoforms, nidogens, or heparan sulfate proteoglycans, to BBB integrity has not been extensively studied (Engelhardt and Sorokin 2009). Initial evidence concerning the contribution of ECM proteins to BBB characteristics has come from studies showing that endothelial barrier properties increase in the presence of astrocyte- or pericyte-derived ECM proteins, and that the enzymatic disruption of ECM proteins decreases BBB characteristics in vivo (Rosenberg et al. 1993; Hartmann et al. 2007). Thus, the specific role of individual ECM proteins in the development or maintenance of barrier characteristics of brain endothelial cells awaits further studies.

One specific molecule of interest in this context is the heparan sulfate proteoglycan agrin, which has been found to accumulate extensively in the basement membrane of the brain microvasculature (Kroger and Mann 1996). A potential role for agrin in BBB development is suggested by the observation that it accumulates around brain microvessels during chicken and rat embryonic development (Barber and Lieth 1997). In contrast, the loss of agrin expression has been shown to be correlated with the loss of expression of junctional proteins in brain vessels of human glioblastoma specimens or with the enhanced BBB leakiness to blood-borne molecules in brains of patients with Alzheimer’s disease (Rascher et al. 2002; Berzin et al. 2000), or after global cerebral ischemia (Baumann et al. 2009) supporting a potential role of agrin in BBB maintenance.

Agrin is a large extracellular multidomain heparan sulfate proteoglycan originally isolated from the electric organ of the marine ray *Torpedo californica*. It was identified as organizing the aggregation of skeletal-muscle-expressed acetylcholine receptors (AChR) beneath the nerve terminal (Nitkin et al. 1987). Subsequently, agrin was found to be released by motor neurons from their terminal axons, where it serves as a critical organizer of postsynaptic differentiation (Gautam et al. 1996). In contrast, little is known about the role of agrin at sites other than the neuromuscular junction, including its role in the brain. The high expression level of agrin in the brain during embryonic development has suggested a role in the formation of synapses other than those at the neuromuscular junction. Indeed, evidence has been presented that agrin plays a role in CNS synapse formation (Ksiazek et al. 2007). In addition, agrin localization is observed in all basement membranes of the CNS including those of the retina (Kroger and Schroder 2002).

More recent studies have provided evidence for the existence of multiple, alternatively spliced isoforms of agrin with various specificities and signaling capabilities (Bezakova and Ruegg 2003). An alternative first exon can be used at the amino terminal region to generate agrin isoforms that do not contain the N-terminal (NtA) domain and are not secreted, but instead immobilized as type II transmembrane proteins. Two agrin isoforms containing the laminin-binding NtA domain at the N-terminus can be distinguished based on the presence or absence of a seven-amino-acid insertion (Denzer et al. 1995). The insertion of seven amino acids at this N-terminal splicing site seems to be specific for the neuronal chicken agrin (Denzer et al. 1995). In addition, the COOH-terminal region of agrin splice sites (referred to as A or B in the chicken and y or z in rodents) accepts peptide inserts of 4 or 8/11/19 amino acids, respectively (Bezakova and Ruegg 2003). Importantly, the distinct receptor-binding properties of the agrin isoforms are attributed to the alternative splicing sites A/y and B/z at the C-terminus of agrin (Ruegg et al. 1992; Kroger and Schroder 2002). Thus, the secreted neuronal agrin that mediates the aggregation of postsynaptic proteins displays the insertions of 7, 4, and 8 amino acids in the A/y and B/z sites and is referred to as agrin 748. In contrast, amino acid inserts at the A/y and B/z sites negatively regulate binding of agrin to the agrin-binding protein α-dystroglycan (α-DG) on astrocytic endfeet (Scotton et al. 2006). Thus, unsurprisingly, the non-neuronal agrin isoform without a peptide insert in the A/y and B/z site (agrin 000) has been reported to be most prominent in the vascular basement membranes of the CNS (Kroger and Schroder 2002).

As the role of agrin localized in brain microvascular basement membranes remains unclear, we have asked, in the present study, whether this heparan sulfate proteoglycan has a direct impact on the barrier characteristics of brain endothelial cells in vitro and in vivo. The culture of the brain endothelioma cell line bEnd5 on agrin 000 or agrin 748 significantly enhances the barrier characteristics of bEnd5 monolayers. Decreased permeability of bEnd5 monolayers is accompanied by the enhanced localization of vascular endothelial (VE)-cadherin, β-catenin, and zonula occludens (ZO)-1, but not of the TJ proteins claudin-5 and occludin, in cell-to-cell junctions of bEnd5. Expression levels of VE-cadherin, β-catenin, and ZO-1 have been found to be unchanged indicating that agrin stabilizes these proteins at cell-to-cell junctions rather than inducing their enhanced expression. To confirm these in vitro findings, we have analyzed the integrity of the BBB in agrin-deficient mice, the survival of which is a result of a muscle-wide expression of a miniaturized form of neural agrin that rescues focal neuromuscular innervation (c-mag_B8_//agrn^−/−^ mice; Lin et al. 2008). Indeed, brain microvessels in c-mag_B8_//agrn^−/−^ mice that lack agrin in the vascular basement membranes also show decreased junctional staining for VE-cadherin. Thus, our data support the notion that the heparan sulfate proteoglycan agrin contributes to barrier characteristics of brain endothelial cells by stabilizing junctional localization of the adherens junction (AJ) proteins VE-cadherin, β-catenin, and ZO-1.

## Materials and methods

### Antibodies

The following primary antibodies were used for immunohistochemistry (IHC) and immunofluorescence (IF) staining: monoclonal mouse anti-chicken agrin antibodies 5B1 (1:500) and C3 (1:200; Reist et al. 1987), polyclonal rabbit anti-mouse agrin serum 204 (1:1000; Eusebio et al. 2003), polyclonal rabbit anti-claudin-5 (1:100; 34–1,600, Zymed), polyclonal rabbit anti-ZO-1 (1:100; 61–7,300, Invitrogen), polyclonal rabbit anti-ZO-1-associated Y-box factor (ZONAB, Mid; 1:150; 40–2,800, Invitrogen), polyclonal rabbit anti-ZO-2 (1:100; 71–1,400, Zymed), polyclonal rabbit anti-occludin (1:50; 71–1,500, Zymed), polyclonal rabbit anti-β-catenin (1:2000; C2206, Sigma), monoclonal rat anti-VE-cadherin (11D4.1, hybridoma supernatant; Gotsch et al. 1997; kindly provided by Prof. Dietmar Vestweber, Münster, Germany), monoclonal rat anti-JAM-A (BV12; Bazzoni et al. 2005; kindly provided by Prof. Elisabetta Dejana, Milan, Italy), polyclonal rabbit anti-laminin (1:1000 for IHC, 1:4000 for IF; Z0097, DakoCytomation), polyclonal rabbit anti-caveolin (1:450; 610,059, BD Transduction Laboratories), biotinylated polyclonal goat anti-mouse IgG (1:200; BA9200, Vector). The following isotype controls were applied: normal rabbit IgG (AB-105-C, R&D Systems), purified mouse IgG1 (400,101, Biolegend), normal rabbit serum (C125B, Serotec), monoclonal rat anti-human CD44 (9B5, hybridoma supernatant), and purified rat IgG2a (553,927, Pharmingen).

For IF staining, the secondary antibodies DyLight-488-conjugated Streptavidin (1:500; 016-480-084, Jackson), Alexa-Fluor-488 polyclonal goat anti-rabbit IgG heavy and light (H + L), polyclonal goat anti-rat IgG H + L (1:200; Molecular Probes, Invitrogen), AMCA-conjugated AffiniPure F (ab’) 2 fragment goat anti-rabbit IgG H + L (1:100; Jackson, ImmunoResearch), Cy3-conjugated AffiniPure polyclonal goat anti-rat IgG H + L, goat anti-rabbit IgG H + L, and polyclonal donkey anti-mouse IgG H + L (1:200; Jackson, ImmunoResearch) were employed.

For IHC, secondary biotinylated polyclonal goat anti-rabbit IgG and anti-rat IgG antibodies from the Vectastain ABC kits (Vector PK 6104 and PK 6101) were used for primary antibody detection.

### Cells

Human embryonic kidney (HEK) cells expressing the secreted non-neuronal chicken agrin A0A0B0 (agrin 000) or the secreted neuronal chicken agrin A7A4B8 (agrin 748) isoforms and their culture conditions were as previously described in detail and were used unchanged in the present study (Kroger 1997). HEK cells were seeded on laminin (from Engelbreth-Holm-Swarm sarcoma, Roche), and after 7 days of culture expression, the secretion and deposition of agrin on laminin was analyzed by IF staining of the HEK cells before or after lysis with 0.3 % (v/v) Triton X-100 (Fluka) in phosphate-buffered saline (PBS) or by Western blot analysis.

The polyoma middle T oncogen transformed mouse brain endothelioma cell line bEnd5 was established by Werner Risau, with its endothelial characteristics, including high purity and contact-inhibition, described in great detail by Reiss et al. (1998). More recently, we have provided a more detailed analysis of bEnd5 as an in vitro BBB model (Paolinelli et al. 2013; Steiner et al. 2011).

The isolation, culture, and characterization of primary mouse brain microvascular endothelial cells (pMBMECs) were performed exactly as previously described in detail (Coisne et al. 2005). We and others have described the characteristics of pMBMEC cultures and consider them to be excellent in vitro BBB models of high endothelial purity forming monolayers that establish a tight permeability barrier (Coisne et al. 2005, 2006; Steiner et al. 2011). Exactly as described before, pMBMECs were cultured on Matrigel containing a mixture of ECM proteins (mostly laminins) extracted from the Engelbreth-Holm-Swarm (EHS) mouse sarcoma tumor. To verify the lack of agrin in the Matrigel matrix, we performed Western blot and IF staining of the matrix.

### Mice

The chicken mini-agrin transgenic (c-mag_B8_), agrin-deficient (agrn^−/−^) mice have been previously described (Lin et al. 2008) and are referred to as c-mag_B8_//agrn^−/−^ mice or rescued agrin knock-out mice below. The transgenic animals express the gene for the chicken mini-agrin, consisting of the 25 kDa N-terminal and 21 kDa C-terminal fragment of the secreted full-length neuronal chicken agrin protein, which include the laminin-binding and acetylcholine-receptor-aggregating domains, respectively, under the muscle creatine kinase (MCK) promoter to restore neuromuscular junction formation and to prevent perinatal death (Moll et al. 2001). All animal procedures were performed in accordance with Swiss legislation concerning the protection of animals and were approved by the Amt für Landwirtschaft und Natur, Veterinärdienst, Sekretariat Tierversuche (Department for Agriculture and Nature, Veterinary Service, Registry for Animal Experimentation) of the Kanton Bern.

### IF staining of brain endothelial cells

IF staining was performed exactly as described previously (Steiner et al. 2011) and was analyzed by using a Nikon Eclipse E600 microscope equipped with a digital camera. Images were further processed with NIS Elements software. To analyze the gray values of the junction-associated IF signal, ImageJ software 1.42q was used. To this end, random lines were drawn over a micrograph of the cell monolayer at original magnification, and the measured peaks of the gray values clearly associated with the cell-cell junctions were noted (Supplementary Fig. 1). For the calculation of the mean gray value per image, 8 gray values of cell-cell junctions randomly distributed over the entire micrograph were selected in a blinded manner. This procedure was repeated for 5 images per condition for each single experiment. The gray value means of each single experiment, representing 40 measured gray values of cell-cell junctions per condition, are represented in the figures below by a symbol and a line, the latter showing the mean values of all experiments performed.

Lipid raft staining was performed with the cholera toxin subunit B (CTx-B) conjugated to Alexa-Fluor-488 (1:100; C-34775, Molecular Probes) for 1 h at room temperature, followed by fixation in 1 % (w/v) paraformaldehyde in PBS (PFA/PBS) for 10 min at room temperature, staining of the cell nuclei with 4′6-diamidino-5-phenylindole (DAPI; Applichem), and mounting of the specimens in Mowiol (Calbiochem).

### IF/IHC staining of tissue sections

IF and IHC staining of mouse brain tissue sections was performed exactly as described by our group previously (Deutsch et al. 2008). The sections were analyzed by using a Nikon Eclipse E600 microscope equipped with a digital camera. Images were further processed with NIS Elements software. For optical sectioning and three-dimensional (3D) reconstruction, 10-μm-thick cryosections were stained as described previously, and 10 stacks/vessel every 400 nm were photographed via an AxioObserver Z1 microscope equipped with an ApoTome and a monochrome charge-coupled device camera (Carl Zeiss, Switzerland).The sections were further analyzed with AxioVision (Carl Zeiss) software.

### Fluorescence-activated cell sorting analysis of bEnd5 cells

Fluorescence-activated cell sorting (FACS) analysis for cell surface proteins of bEnd5 cells was performed exactly as described previously (Engelhardt et al. 1997; Reiss et al. 1998). Cell surface expression of VE-cadherin was determined by using the rat-anti-mouse monoclonal antibodies 11D4 and BV13. Data were analyzed by using BD FACSCalibur and FlowJo Software (TreeStar).

### Gel electrophoresis and Western blot

Brain endothelial cells were washed three times with ice-cold PBS, scratched off the culture dish surface, and harvested in 2× sample buffer (Laemmli Buffer, 200 mM dithiothreitole). The samples were heated for 3 min at 95 °C, separated on a polyacrylamide gel, and blotted onto nitrocellulose (Whatman Protran transfer membrane, 0.2 μM pore size). To analyze the protein content of the brain, one cerebral hemisphere per animal was homogenized in 10 volumes (w/v) of ice-cold RIPA buffer (50 mM TRIS pH 7.5, 150 mM NaCl, 0.5 % Na-deoxycholate, 0.1 % SDS, 1 % NP-40) containing protease inhibitors (Complete Mini EDTA free protease inhibitor cocktail tablets, Roche). After a 5-min centrifugation step at 3000*g*, the supernatant was collected, and the protein concentration was determined with a BCA protein assay kit (Pierce). Of the total protein per sample, 30 μg was mixed with 2× sample buffer, heated for 3 min at 95 °C, and loaded onto a polyacrylamide gel. After the blocking of the nitrocellulose membrane in PBS/2 % (w/v) bovine serum albumin (fraction V, Applichem) or PBS/5 % milk for 20 min at room temperature, the membrane was incubated with the following primary antibodies overnight at 4 °C: mouse anti-β-actin antibody (1:1000; A5316, Sigma), rabbit anti-ZO-1 (1:1000; 61–7,300, Invitrogen), rat anti-VE-cadherin (3 μg/ml; 11D4.1, purified), rabbit anti-β-catenin (1:1000; C2206, Sigma). After being washed with PBS, the primary antibodies were detected by goat anti-mouse or anti-rat IRDye 680 or goat anti-mouse or anti-rabbit IRDye 800CW (LI-COR Biosciences), and the membrane was scanned with the Odyssey Infrared imaging system. The intensities of the protein bands analyzed were always normalized to the corresponding β-actin band intensities.

### Quantitative reverse transcription plus the polymerase chain reaction

Quantitative real-time reverse transcription plus the polymerase chain reaction (RT-PCR; of 1 ng cDNA per reaction in triplicate) was performed exactly as previously described (Lyck et al. 2009). In brief, cellular RNA was extracted from bEnd5 after 24 h in culture with the RNAready kit (BioDiagnostik) according to the manufacturer’s protocol. cDNA was synthesized from 60-80 ng RNA by using the SuperScript First-Strand Synthesis System for RT-PCR (Invitrogen) with random hexamers. Control cDNA was generated without adding reverse transcriptase. Expression of the genes of interest was analyzed after 40 cycles by using the MESA GREEN qPCR MasterMix Plus for SYBR Assay I Low Rox and the Fast Real-Time PCR System 7,500 or the ViiA7 RT-PCR System (Applied Biosystems). Relative expression values were calculated according to the comparative ∆C_T_ method (RQ = relative expression = 2^-∆CT^, ∆C_T_ = average C_T_ value of target gene-average C_T_ value of endogenous reference gene). s16 ribosomal protein mRNA served as endogenous control. The following primer pairs were used: target ZO-1: sense *tggtgaagtctcggaaaaatg*; reverse *tgctgccaaactatcttgtga*; target VE-cadherin: sense *gttcaagtttgccctgaagaa*; reverse *gtgatgttggcggtgttgt*; target β-catenin: sense *gcagcagcagtttgtggag*; reverse *tgtggagagctccagtacacc*; s16r protein: sense *gatattcgggtccgtgtga*; reverse *ttgagatggactgtcggatg*. The primers were designed to obtain a PCR product at around 80 nucleotides. The C_T_ values for target and reference genes were always below 29 cycles.

### Proliferation assay

The proliferative activity of the cells was measured by the addition of 1 μCi per well ^3^H-thymidine (Amersham, UK) for the last 16 h of culture, and incorporated ^3^H-thymidine was detected with a Liquid Scintillation Counter (LS 5000CE, Beckman).

### Annexin V/propidium iodide staining

Cellular apoptosis and necrosis was determined by staining for Annexin V and propidium iodide (PI) and subsequent measurement by flow cytometry on a FACSCalibur and analysis with FlowJo Version 9.1 software.

### In vivo BBB permeability assay

Eleven c-mag_B8_//agrn^−/−^ mice and nine respective controls (4c-mag_B8_//agrn^+/−^ and 5 c-mag_B8_//agrn^+/+^) were injected intravenously with 1000 μg/animal Cascade blue 3-kDa Dextran (Molecular Probes) and 1000 μg/animal fluorescein isothiocyanate (FITC) 10-kDa Dextran (Molecular Probes). After 15 min, mice were anesthetized with Isoflurane (Baxter) and perfused with 10 ml cold PBS followed by fixation in 10 ml 4 % (w/v) PFA/PBS. Brains and kidneys were removed and snap-frozen in Tissue-TEK (OCT compound, Sysmex Digitana) on dry ice/2-methylbutane (Grogg Chemie). The tissue was stored at −80 °C, and 6-μm-thick cryosections were mounted on Superfrost Plus (Menzel) slides, air-dried, and fixed with 1 % (w/v) PFA/PBS for 10 min at room temperature. The sections were stained according to the protocol for the IF staining of organ sections and were directly analyzed under the microscope.

### Statistical analysis

Statistical significance was analyzed with Prism Version 4.01, employing the unpaired *t*-test or Wilcoxon signed rank test. *P* < 0.05 was considered as being statistically significant.

## Results

### Agrin reduced the permeability of bEnd5 monolayers

**Fig. 1:**
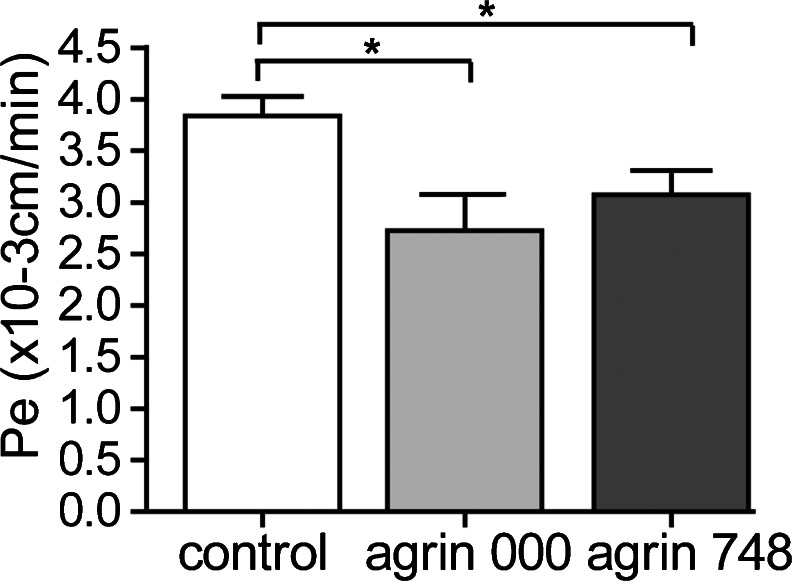
Agrin 000 and agrin 748 reduce permeability of bEnd5 monolayers. The bEnd5 cells were cultured in the presence or absence of chicken agrin 000 or agrin 748, and the permeability to 3-kDa Dextran was measured after 48 h in culture. Bars represent the mean permeability coefficient (*Pe*) values of five independent experiments, each performed in triplicate (± SEM). *P < 0.05

We have previously shown that, in contrast to pMBMECs, the mouse brain endothelioma cell line bEnd5 fails to establish a tight diffusion barrier in vitro (Steiner et al. 2011). Therefore, we first asked whether barrier characteristics of bEnd5 can be improved by growing the cells in the presence of exogenous agrin. As a source for exogenous agrin, we used HEK cells expressing the secreted non-neuronal (agrin 000) or neuronal (agrin 748) chicken agrin isoforms, both containing the laminin-binding NtA domain (Denzer et al. 1995). Non-transfected HEK cells were used to produce control ECM. HEK cells were cultured on laminin, and expression of agrin was detected by IF staining of confluent HEK monolayers at 6–7 days in culture (Supplementary Fig. 2A). The lysis of the HEK cells and subsequent IF staining for agrin detected secreted agrin bound to the laminin matrix (Supplementary Fig. 2B). Additionally, bEnd5 cells were found to express endogenous mouse agrin. Intracellular expression of agrin could be detected in bEnd5 starting at 48 h in culture, whereas deposition of significant amounts of agrin on laminin was only visible after 7 days in culture (Supplementary Fig. 3). To determine whether exogenous agrin influenced barrier characteristics of bEnd5 monolayers, assays were therefore always performed, at the latest, after 48 h of culture. The culturing of bEnd5 cells for 48 h on chicken agrin 000 or agrin 748 resulted in the reduced permeability of bEnd5 monolayers to 3-kDa Dextran when compared with bEnd5 grown on the matrix of control HEK cells. The permeability coefficient (Pe) of bEnd5 for 3-kDa Dextran of 3.8 ×10^−3^ cm/min was reduced to 2.7 ×10^−3^ cm/min on agrin 000 and to 3.1 ×10^−3^ cm/min on agrin 748 (Fig. 1). Thus, the laminin bound exogenously provided agrin isoforms 000 and 748 could both reduce the paracellular permeability of bEnd5 cell monolayers in vitro.

### Agrin increased junctional staining for VE-cadherin, β-catenin, and ZO-1 in bEnd5 cells

**Fig. 2:**
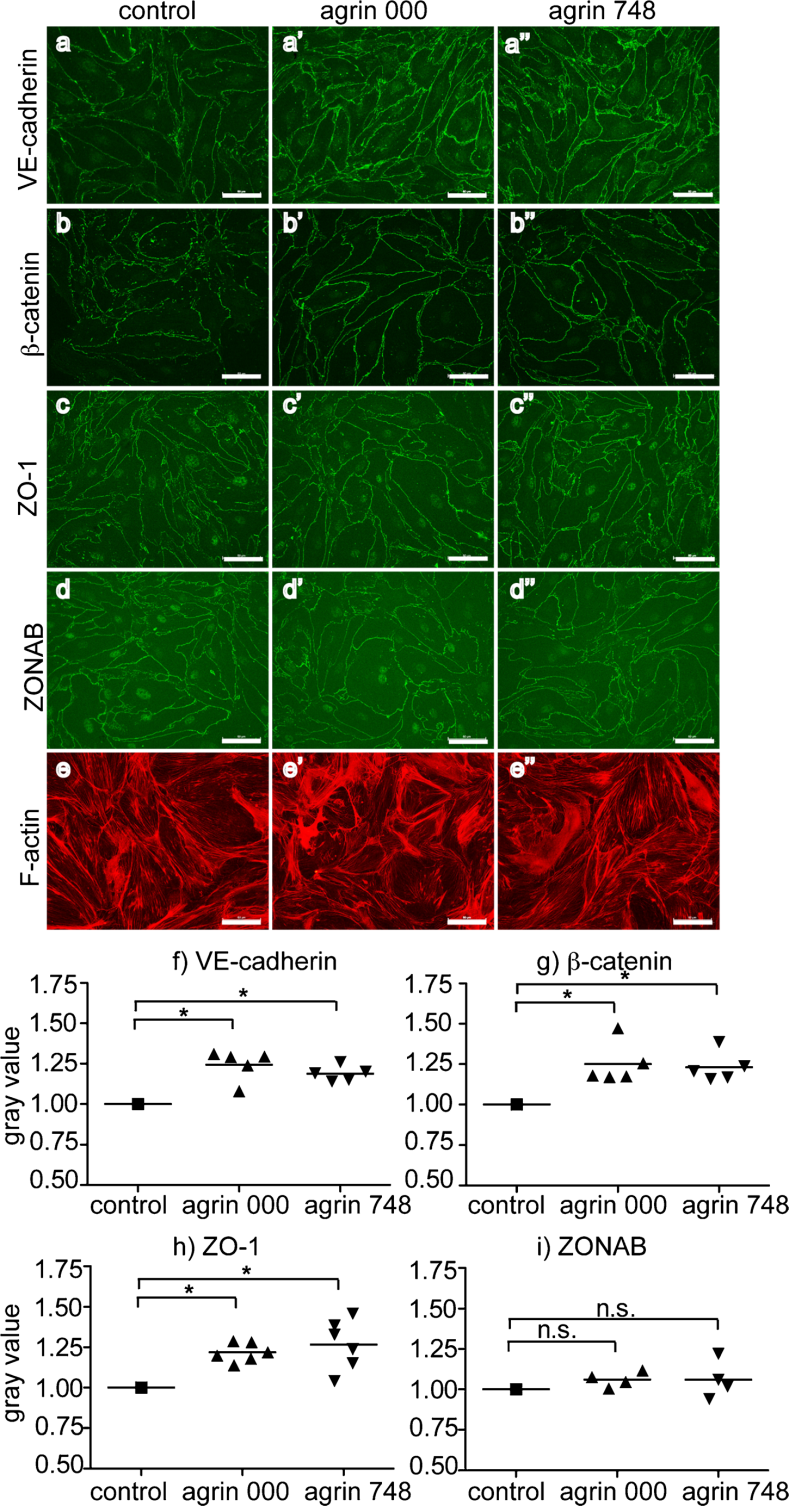
Agrin 000 and agrin 748 increase junctional localization of vascular endothelial cadherin (*VE-cadherin*), β-catenin, and zonula occludens-1 (*ZO-1*) in bEnd5 cells. Immunofluorescence (IF) staining was performed after the culturing of bEnd5 cells for 48 h in the absence or presence of chicken agrin 000 or agrin 748. Cells were stained for VE-cadherin (**a**, **a’**, **a’’**), β-catenin (**b**, **b’** **b’’**), ZO-1 (**c**, **c’**, **c’’**), ZO-1-associated Y-box factor (ZONAB; **d**, **d’**, **d’’**), and F-actin (**e**, **e’**, **e’’**). Representative micrographs from 4–5 independent experiments. *Bar* 50 μm. To quantify the IF signal at the junctions, the gray values of the VE-cadherin (**f**), β-catenin (**g**), ZO-1 (**h**), and ZONAB (**i**) IF signals at the bEnd5 junctions were measured with ImageJ software. *Symbols* represent the mean values of each independent experiment (**f**, **g**, *n* = 5; **h**, *n* = 6; **i**, *n* = 4); the means over all experiments is represented by the *horizontal lines*. Gray values were normalized to the control condition for every independent experiment (*n.s.* not significant)

Agrin-mediated reduction of paracellular permeability of bEnd5 suggested to us an improved junctional architecture of bEnd5. We therefore investigated the cellular localization of several adherens (AJ) and tight (TJ) junction-associated proteins in bEnd5 cells by IF staining. bEnd5 cells grown on control matrix showed the junctional localization of the AJ transmembrane protein VE-cadherin, the AJ-associated cytoplasmic proteins β-catenin and γ-catenin (data not shown), the TJ transmembrane proteins claudin-5 and occludin, and the cytoplasmic scaffolding proteins ZO-1 and ZO-2 (Fig. 2, Supplementary Fig. 4, Supplementary Fig. 5). When bEnd5 cells were grown on agrin 000 or agrin 748, the junctional IF signals for VE-cadherin, β-catenin, and ZO-1 increased when compared with those in bEnd5 cells grown under control conditions (Fig. 2, Supplementary Fig. 4). In contrast, junctional IF staining for claudin-5, occludin, and ZO-2 remained unchanged in bEnd5 cells grown in the presence of agrin (Supplementary Fig. 5). Furthermore, the IF signal of the ZO-1-associated nucleic-acid-binding protein ZONAB, which promotes cellular proliferation via accumulation in the cell nucleus, was also not affected (Fig. 2, Supplementary Fig. 4; Balda et al. 2003). An additional nuclear IF-staining signal was detected for ZONAB and ZO-1 in bEnd5 cells grown on agrin and control matrix thereby supporting their role in brain endothelial cell proliferation and gene expression (Balda and Matter 2009). Finally, we could not detect any signs of F-actin cytoskeletal rearrangement in bEnd5 cells grown on agrin compared with control conditions (Fig. 2). To quantify the microscopically observed changes in junctional IF signals, we performed a computer-assisted measurement of the gray values for the respective IF staining at the cellular junctions (Fig. 2, Supplementary Fig. 1). These analyses showed 18–27 % increased junctional gray values for IF staining for VE-cadherin, β-catenin, and ZO-1, when bEnd5 cells were cultured on agrin 000 and agrin 748 as compared with control (Fig. 2). In contrast, the quantification of junctional gray values of the IF signals for ZONAB (Fig. 2), claudin-5, occludin, and ZO-2 (Supplementary Fig. 5) did not reveal any differences thereby confirming the visual microscopic analysis. Thus, bEnd5 cells grown on agrin 000 and agrin 784 showed increased junctional IF staining for VE-cadherin, β-catenin, and ZO-1.

### Agrin stabilizes VE-cadherin, β-catenin, and ZO-1 in bEnd5 cellular junctions

**Fig. 3:**
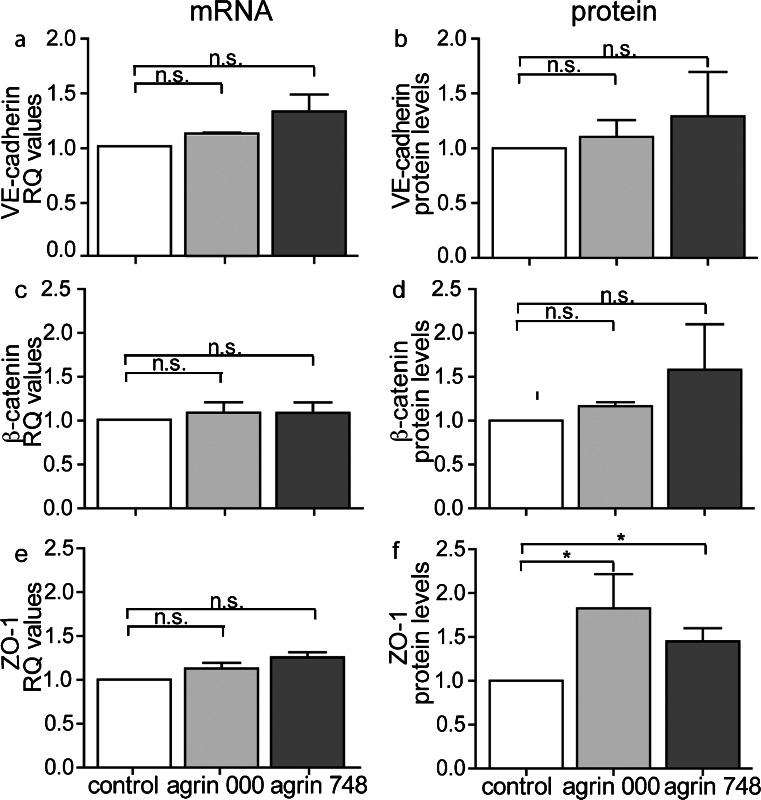
Agrin 000 and agrin 748 do not increase expression levels of VE-cadherin, β-catenin and ZO-1 in bEnd5 cells. **a**, **c**, **e** Quantitative reverse transcription plus the polymerase chain reaction was performed to analyze VE-cadherin, β-catenin, and ZO-1 mRNA levels in bEnd5 cells cultured for 24 h on agrin 000, agrin 748, or under control conditions. Relative expression (*RQ*) values were normalized to the control condition for every independent experiment. *Bars* represent the mean of three independent experiments (± SEM). s16 ribosomal protein mRNA served as endogenous control. **b**, **d**, **f** bEnd5 total VE-cadherin, β-catenin, and ZO-1 protein amounts were determined by Western blot analysis after 48 h in culture. Protein levels were normalized to the endogenous control protein β-actin. Values were normalized to the control condition for every independent experiment. *Bars* represent the mean of three (VE-cadherin, β-catenin) and six (ZO-1) independent experiments (± SEM). *P < 0.05 (*n.s.* not significant)

To test whether the increased junctional IF staining for VE-cadherin, β-catenin, and ZO-1 in bEnd5 cells grown on agrin was attributable to the increased expression of these proteins, we performed RT-PCR and Western blot analyses (Fig. 3). As increased junctional IF staining for these proteins was detected after 48 h of culture of bEnd5 cells on agrin, mRNA was isolated from bEnd5 after 24 h in culture, and total protein of bEnd5 cells was collected after 48 h of culture. mRNA levels for VE-cadherin, β-catenin, and ZO-1 were found to be unchanged in bEnd5 cells grown on agrin 000 or agrin 748 compared with bEnd5 grown on control matrix showing that the transcriptional regulation of VE-cadherin, β-catenin, and ZO-1 in bEnd5 cells is not influenced by the presence of extracellular agrin (Fig. 3). When analyzing total protein levels for these junctional molecules by Western blot, we detected no difference in the amount of VE-cadherin or β-catenin in bEnd5 cells grown in the presence or absence of agrin (Fig. 3b, d). Similarly, cell surface expression of VE-cadherin as determined by FACS analysis was found to be unaltered (Supplementary Fig. 6). In light of the recent observation that E-cadherin molecules can also be localized in the contact-free plasma membrane throughout the epithelial cell (Borghi et al. 2012), these observations suggest that agrin leads to the redistribution of VE-cadherin from contact-free plasma membrane domains into the cell-cell contacts of bEnd5. In contrast, bEnd5 cells, especially when grown on agrin 000 but also on agrin 784, showed significantly increased levels of ZO-1 protein (Fig. 3f). Taken together, these data enable us to conclude that extracellular agrin leads to a difference in the cellular distribution of VE-cadherin, β-catenin, and ZO-1 leading to their accumulation in endothelial junctions of bEnd5 cells. The results also suggest that the presence of extracellular agrin stabilizes the localization of ZO-1, β-catenin, and VE-cadherin in this cellular compartment, an event that in the case of ZO-1, seems to be accompanied by an increase in the half-life of the protein in bEnd5.

### Agrin reduces bEnd5 cell proliferation

**Fig. 4:**
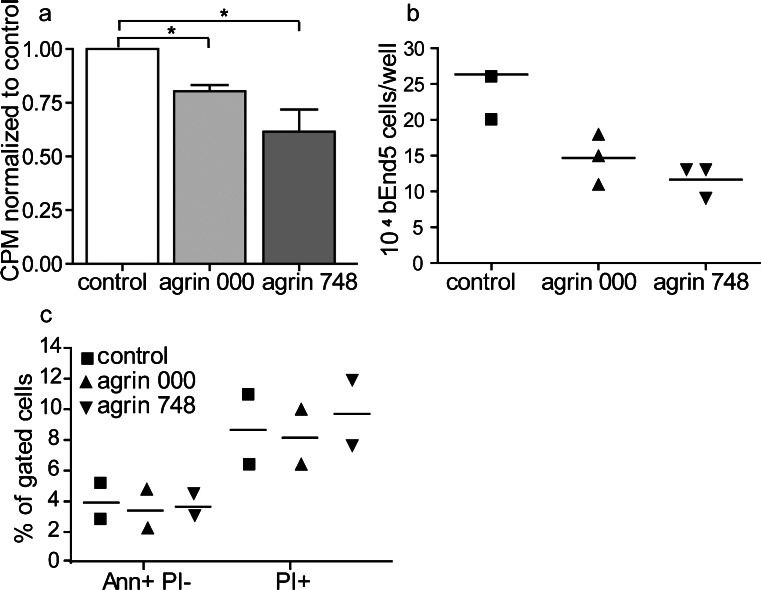
Agrin 000 and agrin 748 reduce the proliferation rate of bEnd5 cells. **a** Cellular proliferation was measured by ^3^H-thymidine uptake after 48 h in culture (*CPM* counts per minute). The values of each experiment were normalized to the respective control condition. *Bars* represent the mean of six independent experiments (± SEM). *P < 0.05. **b** bEnd5 cells were counted after 48 h in culture in the presence of agrin 000, agrin 748, or under control conditions. *Symbols* represent the triplicates of one experiment, and the mean (*horizontal line*) is shown for better visualization. **c** After 48 h, the percentage of apoptotic and necrotic cells was analyzed by Annexin V (*Ann*) and propidium iodide (*PI*) staining. Positive bEnd5 cells were analyzed by flow cytometry. *Symbols* represent values of two independent experiments, and the *horizontal lines* represent the means

Increased accumulation of β-catenin at the cell-cell junctions of bEnd5 cells grown on agrin protein might eventually limit its translocation to the nucleus, where it can act as a transcription factor and upregulate the transcription of cyclin D1 and MYC (Dejana 2004). Inhibition of this activity would indirectly limit the growth of bEnd5 cells. We therefore asked whether bEnd5 cells grown on agrin showed a different cellular proliferation rate when compared with bEnd5 grown in the absence of exogenous agrin. To this end, we measured the proliferation of bEnd5 by analyzing ^3^H-thymidine incorporation into DNA of bEnd5 cells grown on agrin 000, agrin 748, or under control conditions (Fig. 4) and observed a significantly reduced uptake of ^3^H-thymidine, when bEnd5 cells were cultured in the presence of agrin 000 or agrin 748 (Fig. 4). Additional direct counting of bEnd5 cells after 48 h in culture showed a significantly reduced expansion of cell numbers of bEnd5 cells grown on agrin (Fig. 4). To rule out the possibility that reduced bEnd5 cell numbers were attributable to agrin-induced cell death, we performed FACS staining for Annexin V and PI. We could see that agrin neither induced apoptosis (Ann^+^ PI^−^) nor necrosis (PI^+^) of bEnd5 cells under the applied culture conditions (Fig. 4). These data suggested that agrin limited the growth of bEnd5.

### Agrin^−/−^ pMBMECs show reduced junctional localization of VE-cadherin, β-catenin, and ZO-1

**Fig. 5:**
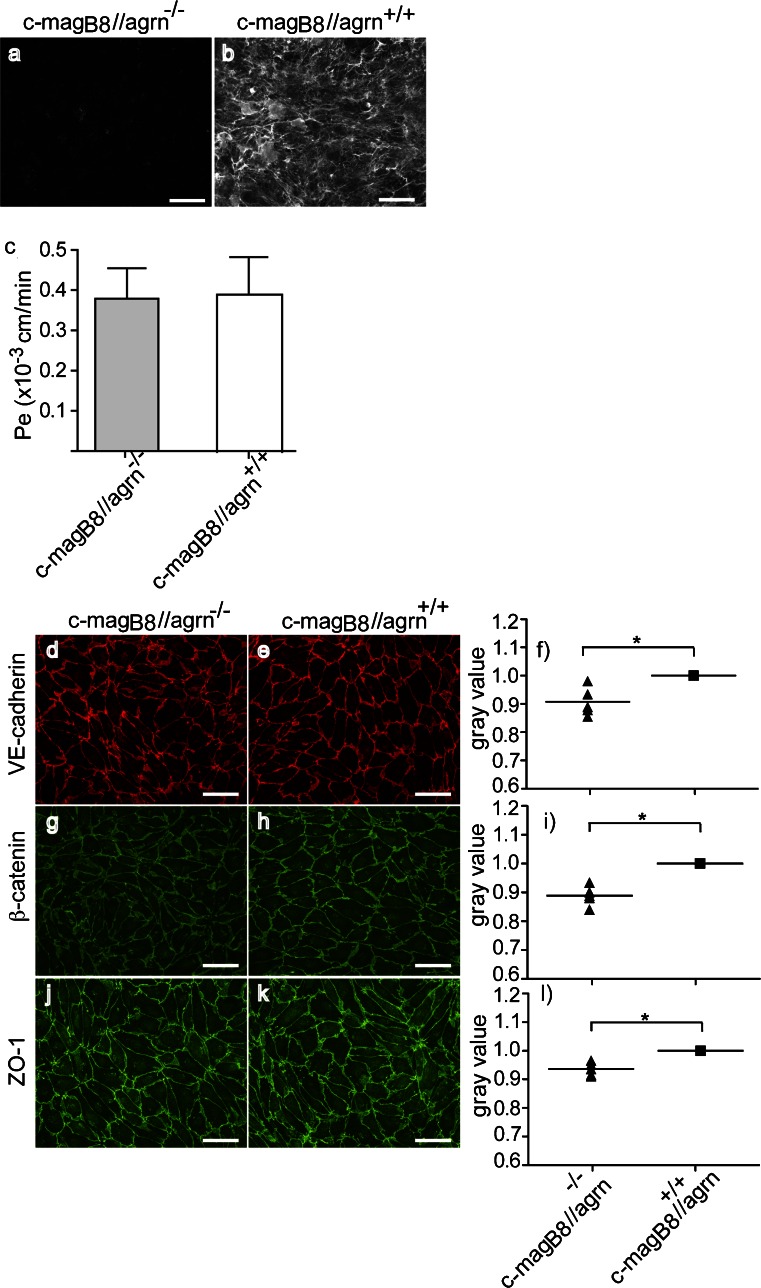
Agrin^−/−^ pMBMECs show reduced junctional localization of VE-cadherin, β-catenin, and ZO-1. **a**, **b** Primary mouse brain microvascular endothelial cells (pMBMECs) were isolated from rescued agrin knock-out (c-mag_B8_//agrn^−/−^) or control littermates (c-mag_B8_//agrn^+/+^), and after 7 days in culture, immunofluorescence staining for agrin was performed. The presence of the extracellular matrix protein agrin is visible by specific binding of the fluorochrome-labeled anti-agrin antibody in control cells (*white signal* in **b**), and the absence of this binding on agrin-knock-out endothelial cells (no fluorescence signal in **a**). Micrographs from one representative experiment out of five are shown. *Bar* 50 μm. **c** Permeability of agrin-deficient (c-mag_B8_//agrn^−/−^) and control pMBMECs (c-mag_B8_//agrn^+/+^) was measured after 7 days in culture for 3-kDa Dextran. *Bars* represent the mean of seven independent experiments (± SEM). **d–i** After 7 days, pMBMECs isolated from rescued agrin knock-out (c-mag_B8_//agrn^−/−^) or control littermates (c-mag_B8_//agrn^+/+^) were stained for VE-cadherin (**d**, **e**), β-catenin (**g**, **h**), and ZO-1 (**j**, **k**), and the junctional IF signal quantified by measuring the gray values at the junctions (**f**, **i**, **l**). Micrographs of one representative experiment out of five are shown. *Bars* 50 μm. The gray values of the knock-out cells were normalized to value of the control cells, which was set to 1. Each *symbol* represents the mean gray value of all five independent experiments, and the *horizontal lines* indicate the means of all experiments performed. *P < 0.05

To further determine the role of agrin in stabilizing VE-cadherin, β-catenin and ZO-1 in brain endothelial junctions, we next asked whether the complete absence of agrin expression in brain endothelial cells disturbed the junctional localization of those molecules. To this end, we isolated pMBMECs from agrin^−/−^ mice that expressed a chicken mini-agrin transgene under the muscle creatine kinase promoter (c-mag_B8_//agrn^−/−^; Lin et al. 2008) and from transgenic control littermates (c-mag_B8_//agrn^+/+^). Immunostaining for agrin confirmed positive staining on pMBMECs isolated from c-mag_B8_//agrn^+/+^ mice and an absence of agrin immunostaining on pMBMECs of c-mag_B8_//agrn^−/−^ mice as expected (Fig. 5). Interestingly, agrin^−/−^ pMBMECs showed reduced junctional IF staining of VE-cadherin, β-catenin, and ZO-1 (Fig. 5, Supplementary Fig. 7) confirming a role for agrin in stabilizing the junctional localization of those proteins. The additional nuclear IF staining signal detected for ZO-1 and β-catenin in pMBMECs from both c-mag_B8_//agrn^+/+^ and c-mag_B8_//agrn^−/−^ mice, supporting their role in brain endothelial cell proliferation and gene expression (Balda and Matter 2009; Liebner and Plate 2010), remained unchanged. However, agrin^−/−^ pMBMECs grown to confluency over 7 days did not show an increased permeability for 3-kDa Dextran when compared with agrin^+/+^ pMBMECs (Fig. 5). This suggests that compensatory mechanisms, e.g., the complex and continuous TJs established by pMBMECs, can overcome the lack of agrin and the accompanying reduced integrity of AJ in pMBMECs to stabilize barrier characteristics in vitro.

### Agrin stabilizes junctional localization of VE-cadherin in brain microvascular endothelial cells in vivo

**Fig. 6:**
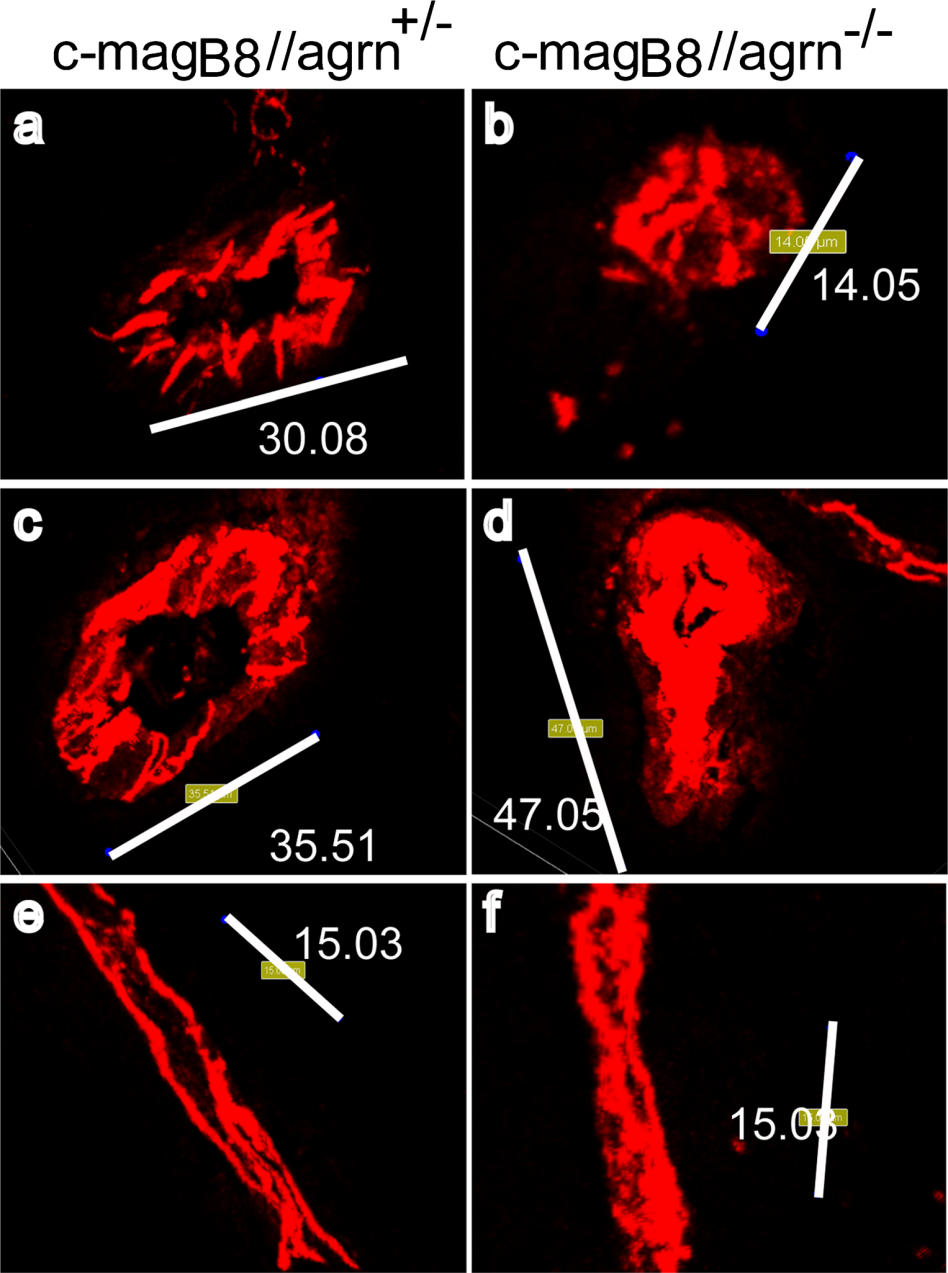
Agrin stabilizes junctional localization of VE-cadherin in brain microvascular endothelial cells in vivo. Three-dimensional reconstruction of VE-cadherin (*red*)-immunostained brain vessels. In each *column*, vessels from three different mice are shown. In c-mag_B8_//agrn^−/−^ mice (**b**, **d**, **f**), VE-cadherin immunostaining is more diffuse and does not display a clear junctional staining pattern compared with the staining observed in vessels of heterozygous control animals (c-mag_B8_//agrn^+/−^; **a**, **c**, **e**). *Indicated numbers* represent the *length* of the *white bar* in micrometers. Representative micrographs of three animals out of four agrin knockout and four control animals are shown

To determine definitively whether the absence of agrin was correlated with reduced junctional localization of AJ molecules in brain vessels in vivo, we investigated whether the junctional localization of VE-cadherin was altered in c-mag_B8_//agrn^−/−^ mice. IF staining, followed by optical sectioning and 3D reconstruction of the endothelial VE-cadherin signal, produced a more diffuse and less circumscribed junctional staining pattern in brain microvessels of c-mag_B8_//agrn^−/−^ mice, when compared with those of heterozygous control mice (c-mag_B8_//agrn^+/−^; Fig. 6). Therefore, our in vivo data further supported the notion that extracellular agrin maintained the integrity of AJs by stabilizing the junctional localization of VE-cadherin. To assess whether the impaired integrity of AJs translated into impaired barrier characteristics of the BBB in vivo, we analyzed the leakage of endogenous immunoglobulin, fibrinogen, and fibronectin and of exogenous 3-kDa Cascade blue and 10-kDa FITC-Dextran across the BBB in female and male c-mag_B8_//agrn^−/−^ mice and c-mag_B8_//agrn^+/+^ control littermates by IF staining and the analysis of brain cryosections. In brain sections of 7 out of 23 c-mag_B8_//agrn^−/−^ mice (age 11–21 weeks, females and males) we found a subset of brain microvessels with deposition of endogenous plasma proteins beyond the laminin positive vascular basement membranes of brain microvessels suggesting focal impairment of the BBB in these mice (Table 1). In brain sections of 3 out of this group of 23 c-mag_B8_//agrn^−/−^ mice we found a subset of brain microvessels with diffusion of intravenously infused 3kDA Cascade blue or 10kDA FITC-dextran, respectively, beyond the laminin positive vascular basement membranes of brain microvessels furthermore underlining focal impairment of the BBB in these mice (Table 1). Combining both methods, we could not determine a correlation of BBB leakiness with age or gender of the mice. Leaky brain microvessels preferentially localized to the cortex, hippocampus, or cerebellum. BBB leakiness was, however, not observed in the remaining 16 c-mag_B8_//agrn^−/−^ mice investigated (Table 1) and was also absent in seven age-matched c-mag_B8_//agrn^+/+^ control mice. The focal nature of the BBB impairment observed in c-mag_B8_//agrn^−/−^ mice, together with the finding that only 29 % of c-mag_B8_//agrn^−/−^ mice showed any signs of focal BBB leakiness, suggests that the lack of agrin in the vascular basement membrane and the resulting impaired AJ integrity do not suffice to impair BBB function. Rather, as previously observed in vitro, compensatory mechanisms, e.g., the unique and complex TJs of the BBB, seem to stabilize the barrier characteristics of the brain microvessels in vivo, and the focal failure of the BBB in a subset of c-mag_B8_//agrn^−/−^ mice might depend on additional pathomechanisms in these rescued agrin knock-out mice.

**Table 1:** Leakage of cerebral microvessels to exogenous and endogenous plasma tracers^a^ in c-mag_B8_//agrn^−/−^ mice depending on gender and age (*BBB* blood-brain barrier)

Tracer	Number of mice with BBB leakage/total mice analyzed	Number of mice with BBB leakage at <6 weeks of age	Number of mice with BBB leakage at 7–21 weeks of age	Number of males with BBB leakage/total males	Number of females with BBB leakage/total females
Exogenous fluorophore-conjugated 3-kDa and 10-kDa Dextrans^a^	3/23	0/2	3/21	3/11	0/12
Endogenous IgG (150–170 kDa) and fibronectin (340 kDa)^a^	7/23	2^b^/2	5/21	4/11	3/12

^a^Leakage of tracers was determined by immunofluorescence staining and analysis of brain cryosections and was defined by the detection of the tracers beyond the laminin-positive vascular basement membranes of brain microvessels

^b^Exclusive leakage of murine IgG

## Discussion

In the present study, we show that the heparan sulfate proteoglycan agrin stabilizes the junctional localization of the AJ proteins VE-cadherin, β-catenin, and ZO-1 in brain endothelial cells in vitro and in vivo without affecting their expression levels in brain endothelial cells. Whereas exogenous agrin reduces the paracellular permeability of brain endothelial cells in vitro, the lack of agrin in primary brain microvascular endothelial cells in vitro and in c-mag_B8_//agrn^−/−^ mice in vivo does not show decreased barrier characteristics. Our observations therefore indicate a novel role of agrin in directly or indirectly stabilizing brain endothelial AJs and thus brain vascular integrity. A potential role for agrin in maintaining BBB characteristics has previously been suggested by Noell et al. (2009); however, their studies focused on the activity of agrin on astrocytes and demonstrated that agrin ensured astrocyte polarity by binding to the astrocyte foot-process anchoring molecule dystroglycan. As we have previously observed agrin immunoreactivity in the endothelial and parenchymal basement membranes of brain microvessels (Wolburg-Buchholz et al. 2009), our aim here has been to determine whether agrin might also exert direct effects on brain endothelial characteristics.

Anticipating potential differences in the biological activities of the secreted agrin isoforms, we cultured bEnd5 cells on both the neuronal (agrin 748) and the non-neuronal (agrin 000) agrin isoforms, which display distinct receptor binding properties because of their alternatively spliced exons coding for isoforms that are different at the C-terminus (Ruegg et al. 1992; Kroger and Schroder 2002). Whereas the neuronal agrin protein is known to bind to LRP4 (Zhang et al. 2008), the non-neuronal agrin isoform binds to α-dystroglycan with high affinity (Meier et al. 1996; Scotton et al. 2006). To our surprise, we could not detect any significant differences between agrin 000 and agrin 748 in enhancing the barrier characteristics of bEnd5 cells. Thus, the endothelial binding domains of agrin and the endothelial ligands for agrin remain to be defined. Possible candidate molecules are endothelial integrins, such as αV-integrin, which has previously been shown to interact with agrin (Martin and Sanes 1997). Alternatively, the glycosaminoglycan side-chains of the heparan sulfate proteoglycan agrin (Winzen et al. 2003) might play a role in the agrin-induced barrier characteristics of brain endothelial cells.

In the present study, we could correlate the agrin-induced reduction of paracellular permeability of bEnd5 to the increased localization of the AJ proteins VE-cadherin and β-catenin and of the scaffolding protein ZO-1 to endothelial cell junctions. Localization of the transmembrane VE-cadherin to endothelial AJs has been reported to be a prerequisite for vascular integrity and junctional maturation of the endothelial cells in peripheral vascular beds (Dejana et al. 2009). The extracellular domains of VE-cadherin establish homophilic adhesive interactions between adjacent endothelial cells. Stabilization of the AJ is achieved by anchoring VE-cadherin to the actin cytoskeleton via its interaction with p120, β-catenin, and α-catenin. The important role for β-catenin in AJ integrity is underlined by the observation that β-catenin-deficient endothelial cells show disorganized AJs and increased paracellular permeability (Cattelino et al. 2003). In addition to the increased junctional localization of the AJ proteins VE-cadherin and β-catenin, agrin also increases the localization of ZO-1 to cell-cell junctions. ZO-1 is a member of the ZO scaffolding protein family, which bind via N-terminal protein-binding domains to transmembrane TJ proteins and via their C-terminus directly to F-actin or other actin-binding proteins (Fanning and Anderson 2009). Combined with the observation that agrin does not influence the cellular distribution of the TJ proteins claudin-5, occludin, and ZO-2, the agrin-induced increased junctional localization of ZO-1 might instead be attributable to its accumulation in AJs. Previous studies have suggested that ZO-1 not only localizes to TJs but can also be present in AJs, most probably via binding to α-catenin (Ikenouchi et al. 2007). The finding that the increased junctional detection of VE-cadherin, β-catenin, and ZO-1 induced by agrin is not accompanied by the increased transcription of these proteins or by the altered cell surface expression levels of VE-cadherin in brain endothelial cells leads us to suggest that agrin instead stabilizes the junctional localization of these proteins. This is further supported by our observation that the junctional localization of VE-cadherin, β-catenin, and ZO-1 is reduced in primary brain endothelial cells deficient for agrin compared with wild-type brain endothelial cells. Confirming previous observations in mice lacking vascular agrin (Rauch et al. 2011), agrin in our present study fails to influence the junctional localization of the TJ proteins claudin-5, occludin, and ZO-2.

In addition to mediating junctional stability, cadherins and their cytoplasmic binding partners are known to be involved in cellular signaling mechanisms. Stabilization of β-catenin in brain endothelial cells has been shown to enhance the maturation of the BBB via the canonical Wnt signaling pathway (Liebner et al. 2008). Cadherins regulate the Wnt signaling pathway by the retention of β-catenin at cellular junctions (Wheelock and Johnson 2003). Upon entering the cell nucleus, β-catenin can function as a transcription factor and can alter the expression of cyclin D-1 (Dejana 2004) and thus promote endothelial cell proliferation (Venkiteswaran et al. 2002). Based on the observation that agrin mediates the junctional stabilization of β-catenin, we have analyzed the proliferation rate of bEnd5 cells in the presence and absence of agrin. In accordance with these previous findings, we have indeed observed decreased proliferation rates in bEnd5 cells when cultured on exogenous agrin.

Interestingly, although the lack of agrin results in the impaired junctional localization of VE-cadherin, β-catenin, and ZO-1 in pMBMECs, this phenotype does not have the impaired barrier characteristics of these cells in vitro. Similarly, although brain microvessels in c-mag_B8_//agrn^−/−^ mice show an impaired junctional localization of VE-cadherin, a focal and heterogeneous defect in BBB integrity has only been observed in less than 30 % of these mice, suggesting that this might instead be attributable to secondary effects in these mice reconstituted for muscular agrin (Lin et al. 2008). This is further supported by previous observations that mice lacking vascular agrin do not develop a leaky BBB in vivo (Rauch et al. 2011). In peripheral vascular beds, AJs have been shown to play a prominent role in regulating vascular permeability, e.g., by regulating the expression and junctional localization of TJ proteins such as claudin-5 (Dejana et al. 2008; Taddei et al. 2008). At the BBB, the expression levels of VE-cadherin are, however, lower than those in peripheral vascular beds (Breier et al. 1996), and conversely, the expression levels of claudin-5 are much higher at the BBB than elsewhere and are indeed critical for the establishment of the barrier functions of CNS microvascular endothelial cells (Nitta et al. 2003). In this context, it is therefore interesting to note that the stabilization of junctional localization of claudin-5 in brain endothelial cells has been reported to rely critically on matrix adhesions of brain endothelial cells via their β1-integrins (Osada et al. 2011); this study has also demonstrated that matrix adhesions at the basal side of brain endothelial cells can affect junctional architecture and thus adhesive contacts at the lateral border between brain endothelial cells. In combination with our present observation that agrin fails to influence the junctional localization of claudin-5, these data lead us to speculate that, especially at the BBB, the reduced junctional localization of VE-cadherin and thus the impaired AJ integrity in the absence of agrin do not suffice to affect TJ integrity and hence compromise the barrier characteristics of the BBB in the absence of additional pathological stimuli in vivo.

The agrin-induced increased junctional localization of AJ proteins in brain endothelial cells is most probably attributable to the aggregating characteristics of agrin. Neuronal agrin has been shown to mediate the aggregation of AChR on myotubes (Gesemann et al. 1995), whereas both neuronal and non-neuronal agrin can stabilize the accumulation of the water channel protein aquaporin-4 to the membranes of astrocyte foot processes (Noell et al. 2007). The ability of agrin to aggregate membrane-associated molecules has been assigned to its ability to concentrate molecules at specialized membrane microdomains such as lipid rafts (Khan et al. 2001). VE-cadherin and ZO-1 have previously been shown to localize to raft-like membrane microdomains suggesting that these microdomains contribute to the clustering of AJ and TJ proteins. This has been further supported by the finding that the knock-down of caveolin-1, a scaffolding protein associated with lipid rafts, leads to the loss of VE-cadherin, β-catenin, and ZO-1 from these detergent-resistant membrane domains (Nusrat et al. 2000; Song et al. 2007). We therefore asked whether the culturing of bEnd5 cells in the presence of exogenous neuronal or non-neuronal agrin would trigger the formation of lipid rafts. By staining bEnd5 cells for the lipid raft marker cholera toxin B (CTx-B) subunit that associates with ganglioside GM1 (Schon and Freire 1989) and additionally for caveolin-1, we could not provide any evidence for the agrin-induced stabilization of VE-cadherin, β-catenin, and ZO-1 in bEnd5 cell-cell junctions by the clustering of lipid rafts. However, we cannot exclude that the clustering of membrane microdomains is difficult to visualize in adherent cells.

In summary, the present study provides the first direct evidence that, beyond its role as a key organizer of postsynaptic differentiation, agrin, by providing matrix adhesions at the basal side of brain endothelial cells, affects the junctional integrity of endothelial AJs and thus contributes to BBB integrity.

## Electronic supplementary material

Below is the link to the electronic supplementary material.

Supplementary Figure 1 Method of gray value image quantification. The *upper row* shows localization of three random *horizontal lines* (*yellow*) drawn by ImageJ software from micrographs taken with NIS Elements software of the immunofluorescently labeled bEnd5 monolayers at original magnification. bEnd5 grown on control matrix (HEKc), or agrin 000 (SK000), or agrin 748 (SK748) are shown. The *lower row* shows the color-coded overlay of the 3 gray value intensity profiles measured by ImageJ along each of the three lines (*Row 1*, *Row 2*, *Row 3*) shown in the micrographs *above*. For calculation of the mean gray value per image, 8 gray values of cell-cell junctions randomly distributed over the entire micrograph in a blinded fashion were then selected. This procedure was repeated for five images per culture condition for each single experiment (GIF 99 kb)

High Resolution Image (TIFF 1094 kb)

Supplementary Figure 2 HEK-cell-produced agrin 000 and agrin 748 remain bound to laminin coated surfaces. **A** Un-transfected human embryonic kidney (HEK) cells (control) and HEK cells stably expressing the secreted laminin-binding agrin isoforms 000 and 748 were cultured for 7 days on laminin and stained for the cell nucleus (DAPI, *blue*) and with the anti-chicken agrin Ab for chicken agrin (*red*). **B** The HEK cells were lysed, and subsequent staining for remaining cell nuclei (DAPI, *blue*) and for agrin deposited on the laminin layer was performed with the anti-chicken agrin Ab (*red*). The *red* IF-signal confirms agrin deposition by the agrin-transfected HEK cells but not the control HEK cells. Representative IF micrographs out of several independent experiments are shown. *Bar* 50 μm (GIF 118 kb)

High Resolution Image (TIFF 2264 kb)

Supplementary Figure 3 Expression of agrin by bEnd5 cells. Mouse brain derived endothelial cells bEnd5 were cultured on laminin and stained for cell nuclei (DAPI, *blue*) and for endogenous mouse agrin (*green*) by using the polyclonal rabbit anti-mouse agrin serum 204 after 48 h (**A**) and 7 days (**B**) in culture. The cells were either permeabilized or non-permeabilized before agrin staining. Therefore, the diffuse agrin IF signal seen in the non-permeabilized images represents extracellular agrin (deposited extracellular matrix), whereas the diffuse IF signal seen on the permeabilized images represents extra- and intra-cellular agrin. Representative micrographs from two experiments are shown. *Bar* 50 μm (GIF 100 kb)

High Resolution Image (TIFF 2387 kb)

Supplementary Figure 4 Agrin 000 and agrin 748 facilitate junctional localization of VE-cadherin, β-catenin, and ZO-1 in bEnd5 cells. To better appreciate the junctional localization of VE-cadherin (**A**), β-catenin (**B**), ZO-1 (**C**), and ZONAB (**D**) after the culturing of bEnd5 cells for 48 h in the absence and presence of chicken agrin 000 or agrin 748, respectively, high power microphotographs are displayed. The images are higher magnification views of the micrographs displayed in Fig. 2. *Bar* 25 μm (GIF 105 kb)

High Resolution Image (TIFF 1400 kb)

Supplementary Figure 5 Agrin 000 and agrin 748 have no influence on the junctional localization of claudin 5, occludin, and ZO-2 in bEnd5 cells. **A–C** bEnd5 cells cultured on agrin 000, on agrin 748, or under control conditions were stained for claudin-5 (**A**), occludin (**B**) and ZO-2 (**C**) after 48 h in culture. Representative micrographs from 3–4 independent experiments are shown. *Bar* 50 μm. **D-F** The gray values of the junctional IF signal of claudin 5 (**D**), occludin (**E**), and ZO-2 (**F**) were measured with ImageJ software. *Symbols* represent the mean values of each independent experiment (**D**, **F**, *n* = 3, **E** *n* = 4), and the overall means are represented by the *horizontal lines*. The gray values were normalized to the control condition for every independent experiment (GIF 196 kb)

High Resolution Image (TIFF 3152 kb)

Supplementary Figure 6 Exogenous agrin does not change cell suface expression of VE-cadherin on bEnd5. bEnd5 cells were scatter-gated on live cells. Overlays show bEnd5 grown on control matrix, agrin 000, or agrin 748 as indicated. From *left to right*, the histograms depict bEnd5 non-stained, stained with isotype control antibody, or stained for VE-cadherin (GIF 16 kb)

High Resolution Image (TIFF 417 kb)

Supplementary Figure 7 Absence of agrin reduces junctional localization of VE-cadherin, β-catenin, and ZO-1 as demonstrated in Agrin^−/−^ pMBMECs. Primary MBMECs isolated from rescued agrin knock-out (c-mag_B8_//agrn^−/−^) or control littermates (c-mag_B8_//agrn^+/+^) were stained for VE-cadherin, β-catenin, and ZO-1. Here, the junctional IF signal is displayed at high-power magnification for better visibility (for quantitative assessment, see Fig. 5). Micrographs are from one representative experiment out of 5. *Bar* 25 μm (GIF 59 kb)

High Resolution Image (TIFF 807 kb)
