# Supplementary figures and images for: The heparan sulfate proteoglycan agrin contributes to barrier properties of mouse brain endothelial cells by stabilizing adherens junctions
Source: Cell Tissue Res. 2014 Aug 9;358(2):465–79. doi: 10.1007/s00441-014-1969-7 (PMC4210653; doi:10.1007/s00441-014-1969-7)

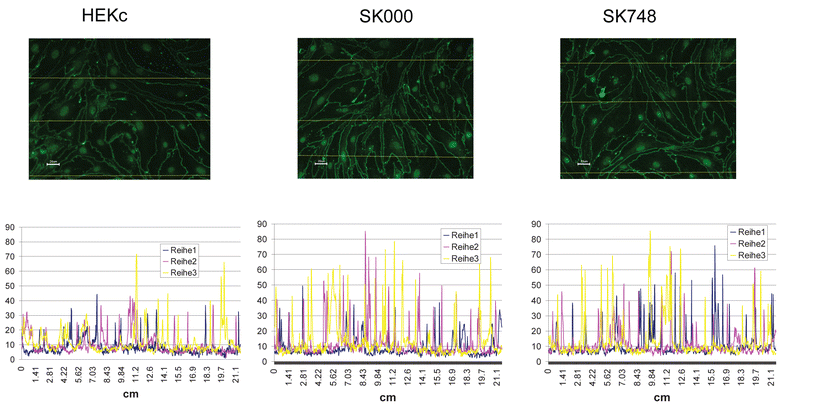

Supplement: Supplementary file 1 — Method of gray value image quantification. The upper row shows localization of three random horizontal lines (yellow) drawn by ImageJ software from micrographs taken with NIS Elements software of the immunofluorescently labeled bEnd5 monolayers at original magnification. bEnd5 grown on control matrix (HEKc), or agrin 000 (SK000), or agrin 748 (SK748) are shown. The lower row shows the color-coded overlay of the 3 gray value intensity profiles measured by ImageJ along each of the three lines (Row 1, Row 2, Row 3) shown in the micrographs above. For calculation of the mean gray value per image, 8 gray values of cell-cell junctions randomly distributed over the entire micrograph in a blinded fashion were then selected. This procedure was repeated for five images per culture condition for each single experiment (GIF 99 kb) [file 441_2014_1969_Fig7_ESM.gif]

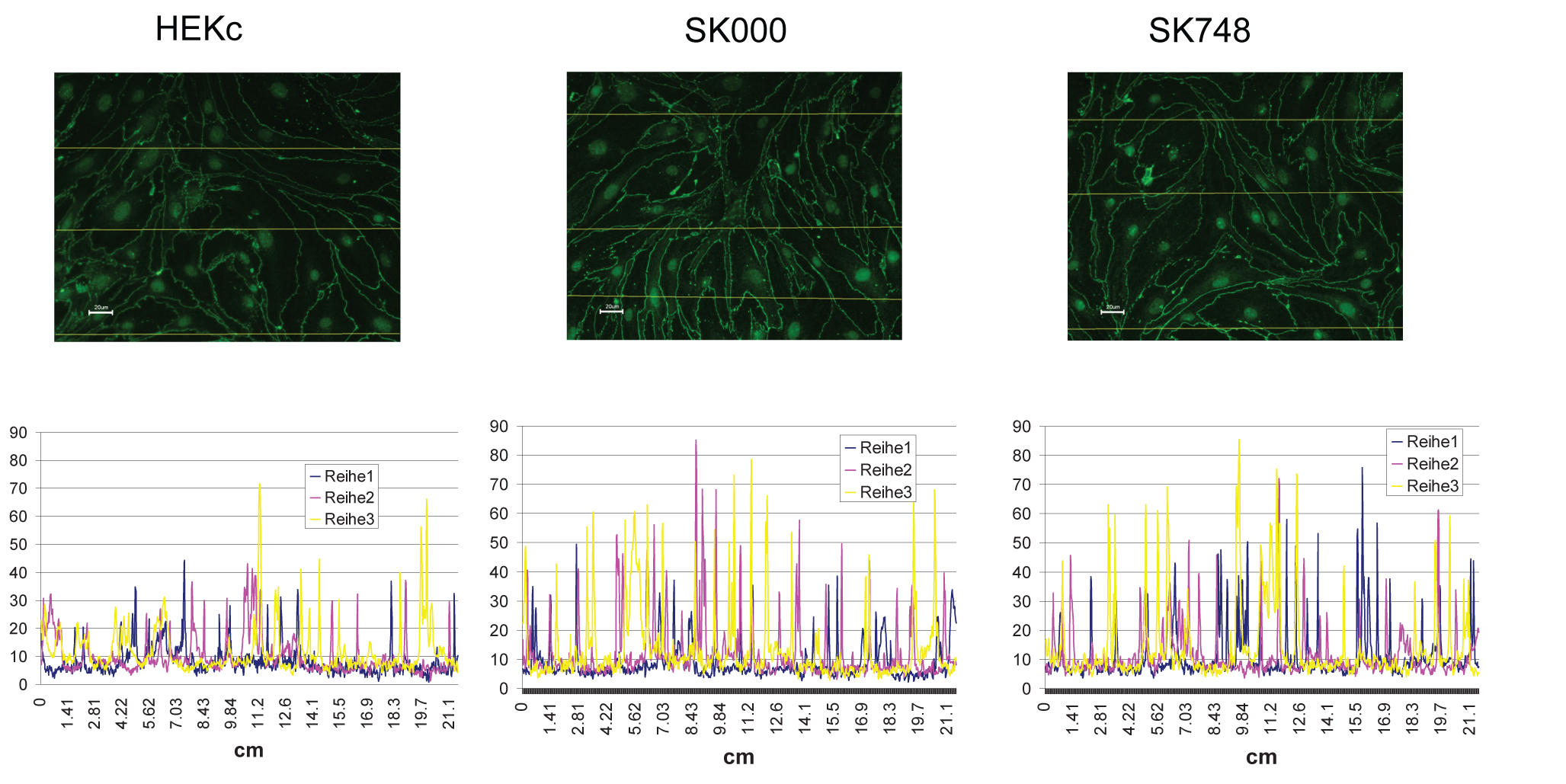

Supplement: Supplementary file 2 — High Resolution Image (TIFF 1094 kb) [file 441_2014_1969_MOESM1_ESM.tif]

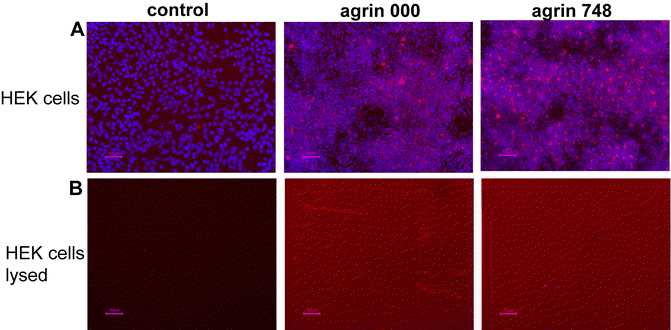

Supplement: Supplementary file 3 — HEK-cell-produced agrin 000 and agrin 748 remain bound to laminin coated surfaces. A Un-transfected human embryonic kidney (HEK) cells (control) and HEK cells stably expressing the secreted laminin-binding agrin isoforms 000 and 748 were cultured for 7 days on laminin and stained for the cell nucleus (DAPI, blue) and with the anti-chicken agrin Ab for chicken agrin (red). B The HEK cells were lysed, and subsequent staining for remaining cell nuclei (DAPI, blue) and for agrin deposited on the laminin layer was performed with the anti-chicken agrin Ab (red). The red IF-signal confirms agrin deposition by the agrin-transfected HEK cells but not the control HEK cells. Representative IF micrographs out of several independent experiments are shown. Bar 50 μm (GIF 118 kb) [file 441_2014_1969_Fig8_ESM.gif]

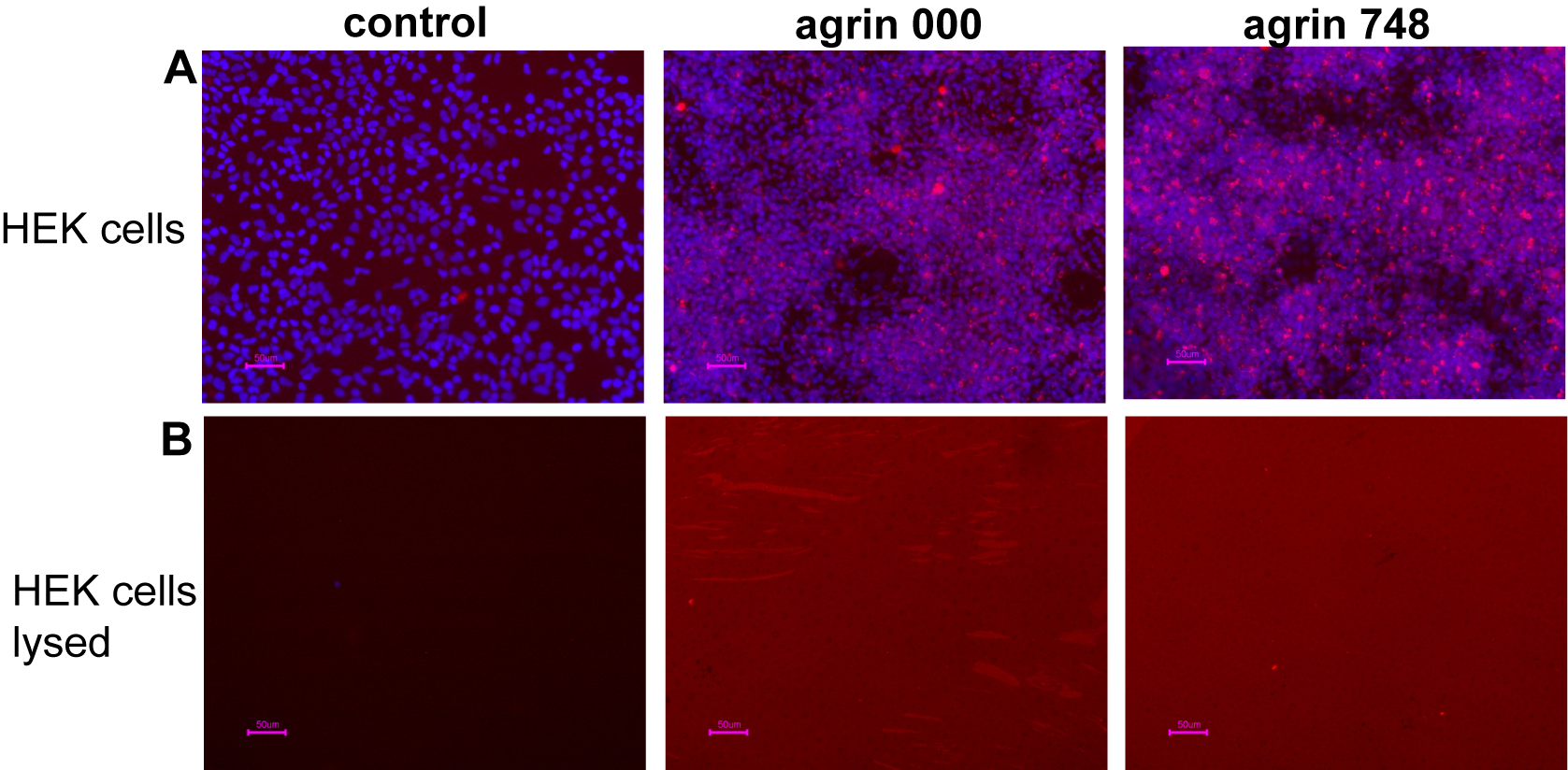

Supplement: Supplementary file 4 — High Resolution Image (TIFF 2264 kb) [file 441_2014_1969_MOESM2_ESM.tif]

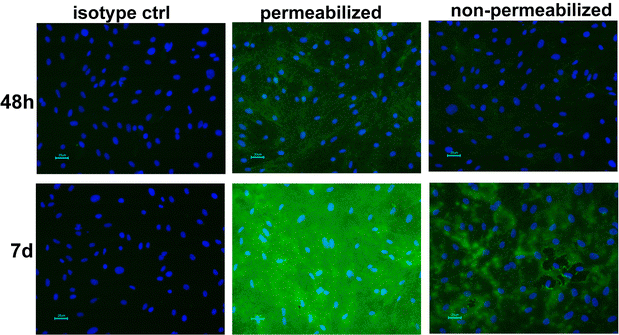

Supplement: Supplementary file 5 — Expression of agrin by bEnd5 cells. Mouse brain derived endothelial cells bEnd5 were cultured on laminin and stained for cell nuclei (DAPI, blue) and for endogenous mouse agrin (green) by using the polyclonal rabbit anti-mouse agrin serum 204 after 48 h (A) and 7 days (B) in culture. The cells were either permeabilized or non-permeabilized before agrin staining. Therefore, the diffuse agrin IF signal seen in the non-permeabilized images represents extracellular agrin (deposited extracellular matrix), whereas the diffuse IF signal seen on the permeabilized images represents extra- and intra-cellular agrin. Representative micrographs from two experiments are shown. Bar 50 μm (GIF 100 kb) [file 441_2014_1969_Fig9_ESM.gif]

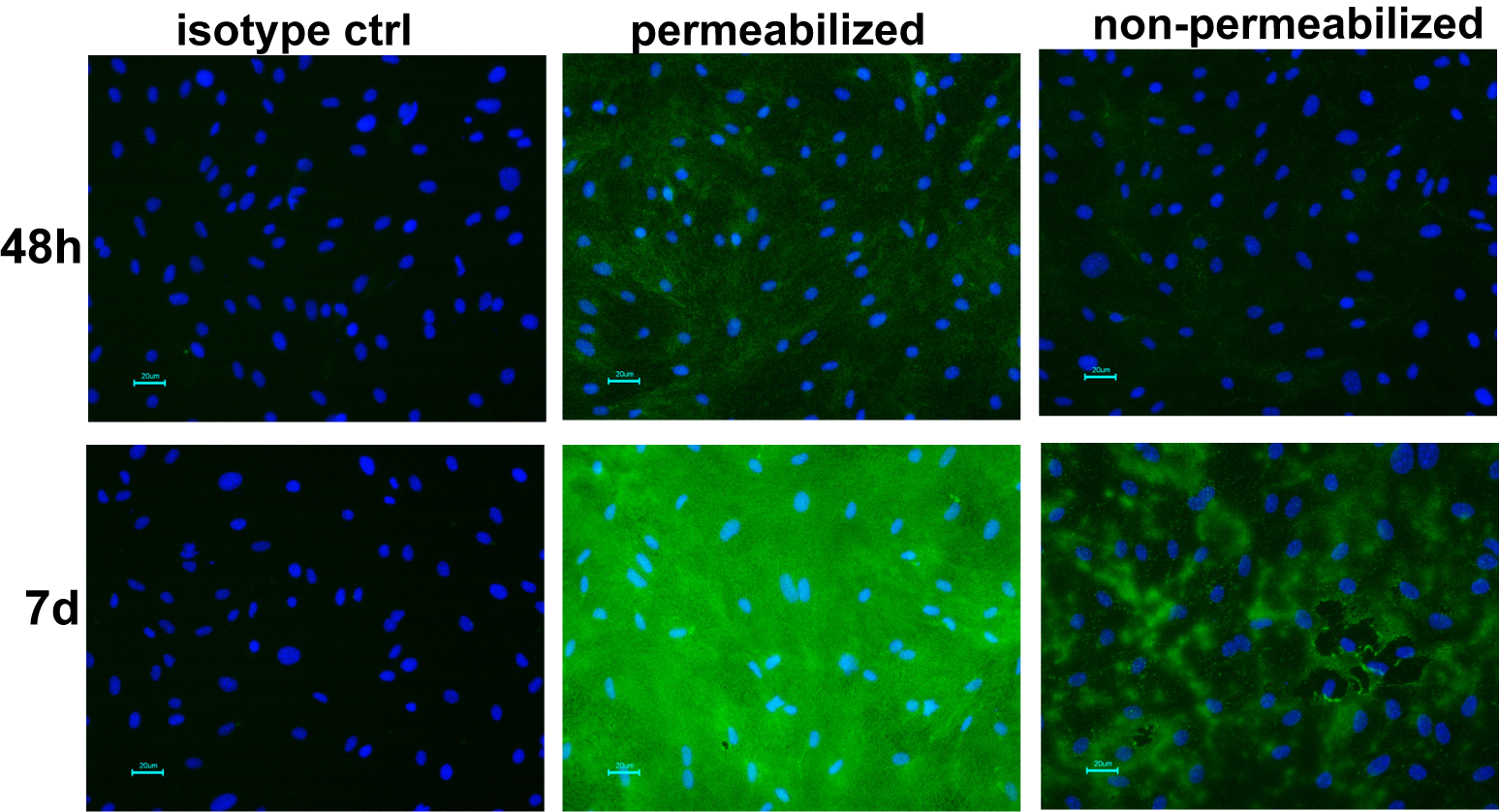

Supplement: Supplementary file 6 — High Resolution Image (TIFF 2387 kb) [file 441_2014_1969_MOESM3_ESM.tif]

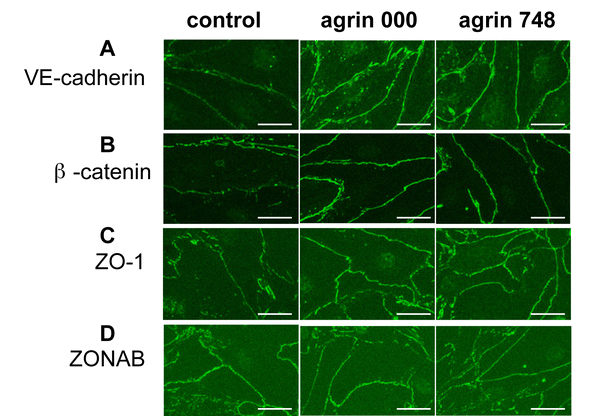

Supplement: Supplementary file 7 — Agrin 000 and agrin 748 facilitate junctional localization of VE-cadherin, β-catenin, and ZO-1 in bEnd5 cells. To better appreciate the junctional localization of VE-cadherin (A), β-catenin (B), ZO-1 (C), and ZONAB (D) after the culturing of bEnd5 cells for 48 h in the absence and presence of chicken agrin 000 or agrin 748, respectively, high power microphotographs are displayed. The images are higher magnification views of the micrographs displayed in Fig. 2. Bar 25 μm (GIF 105 kb) [file 441_2014_1969_Fig10_ESM.gif]

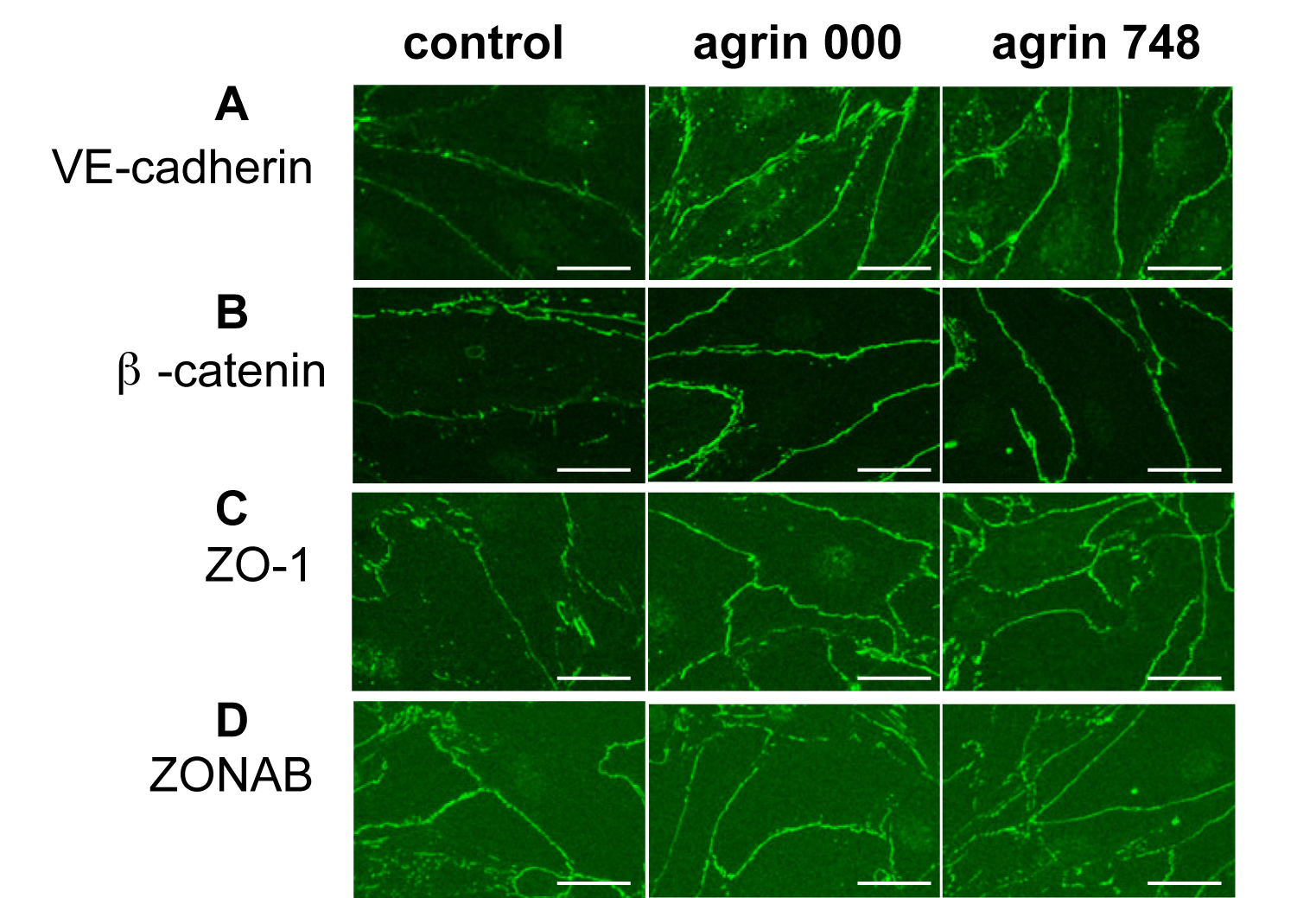

Supplement: Supplementary file 8 — High Resolution Image (TIFF 1400 kb) [file 441_2014_1969_MOESM4_ESM.tif]

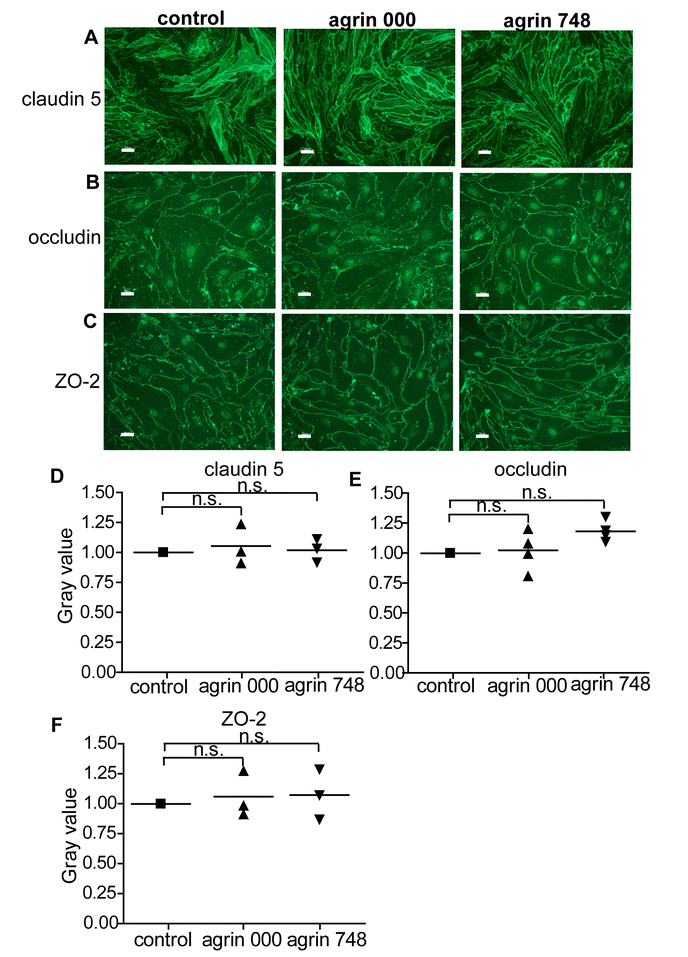

Supplement: Supplementary file 9 — Agrin 000 and agrin 748 have no influence on the junctional localization of claudin 5, occludin, and ZO-2 in bEnd5 cells. A–C bEnd5 cells cultured on agrin 000, on agrin 748, or under control conditions were stained for claudin-5 (A), occludin (B) and ZO-2 (C) after 48 h in culture. Representative micrographs from 3–4 independent experiments are shown. Bar 50 μm. D-F The gray values of the junctional IF signal of claudin 5 (D), occludin (E), and ZO-2 (F) were measured with ImageJ software. Symbols represent the mean values of each independent experiment (D, F, n = 3, E n = 4), and the overall means are represented by the horizontal lines. The gray values were normalized to the control condition for every independent experiment (GIF 196 kb) [file 441_2014_1969_Fig11_ESM.gif]

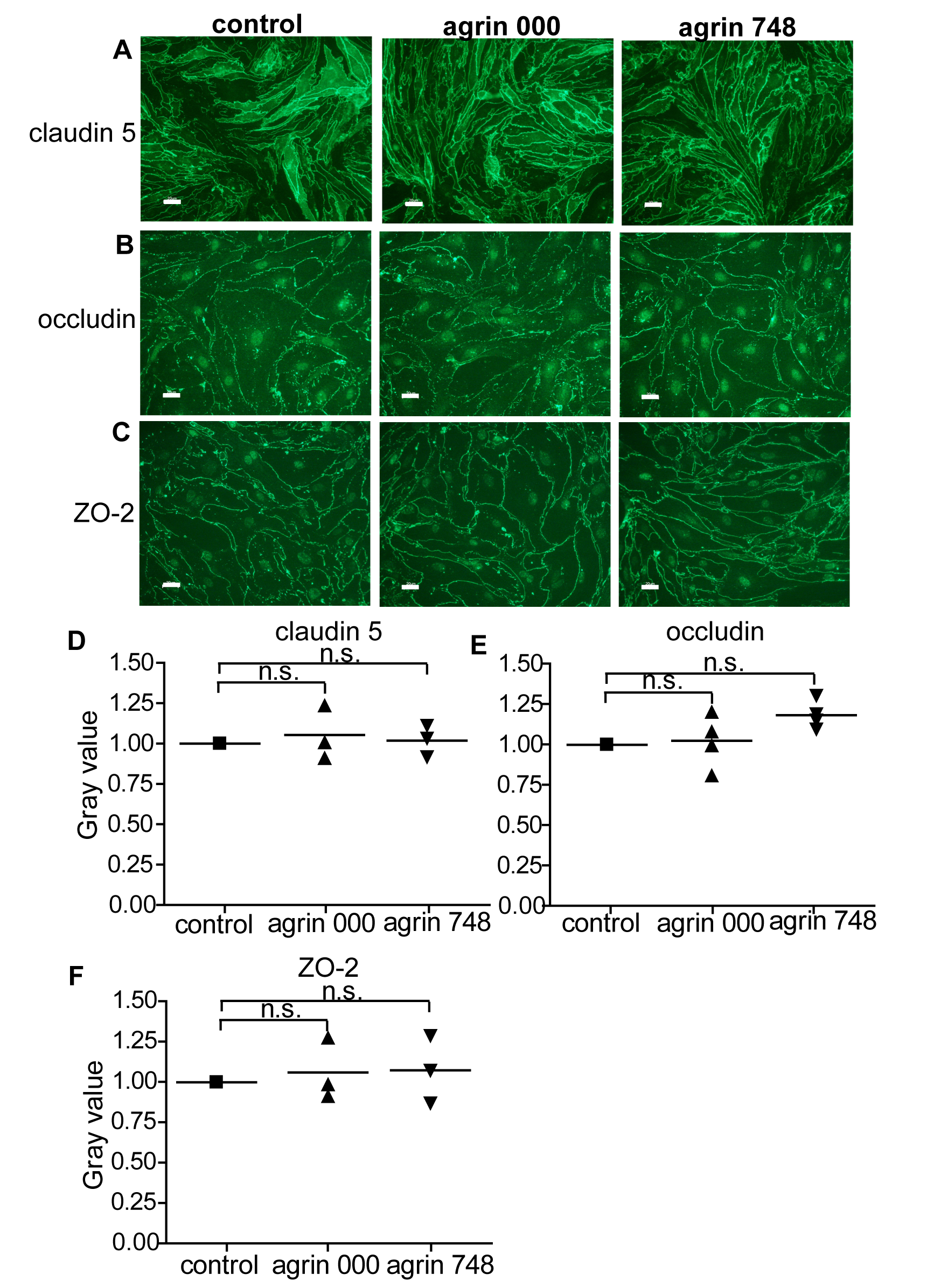

Supplement: Supplementary file 10 — High Resolution Image (TIFF 3152 kb) [file 441_2014_1969_MOESM5_ESM.tif]

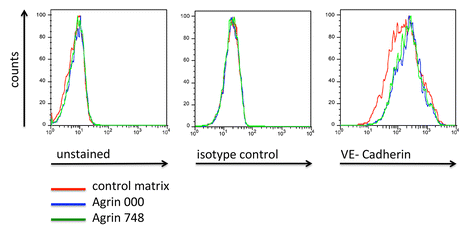

Supplement: Supplementary file 11 — Exogenous agrin does not change cell suface expression of VE-cadherin on bEnd5. bEnd5 cells were scatter-gated on live cells. Overlays show bEnd5 grown on control matrix, agrin 000, or agrin 748 as indicated. From left to right, the histograms depict bEnd5 non-stained, stained with isotype control antibody, or stained for VE-cadherin (GIF 16 kb) [file 441_2014_1969_Fig12_ESM.gif]

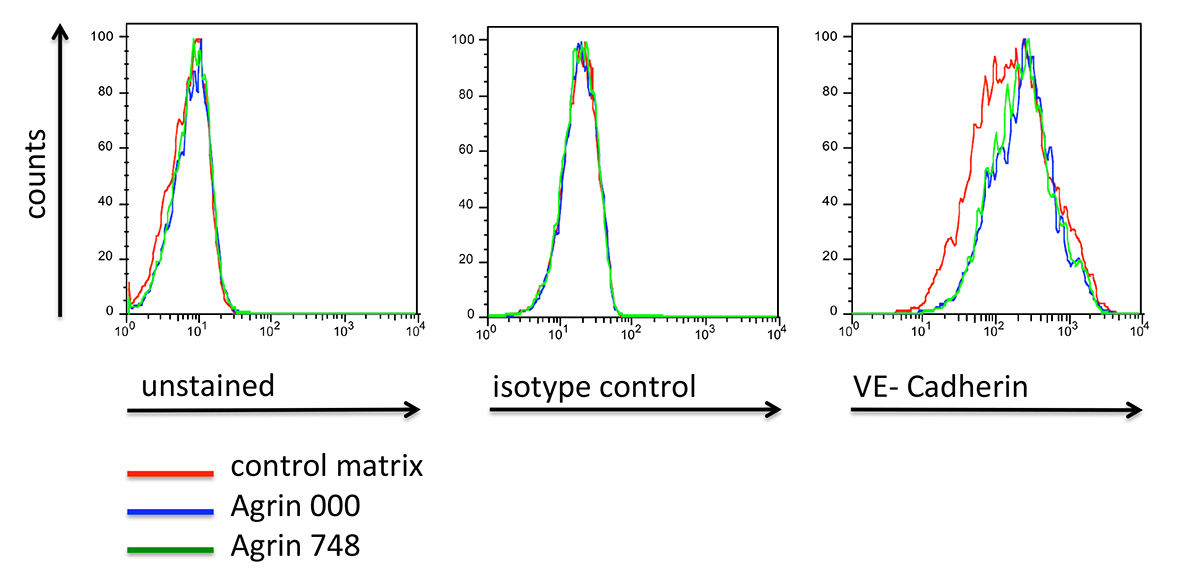

Supplement: Supplementary file 12 — High Resolution Image (TIFF 417 kb) [file 441_2014_1969_MOESM6_ESM.tif]

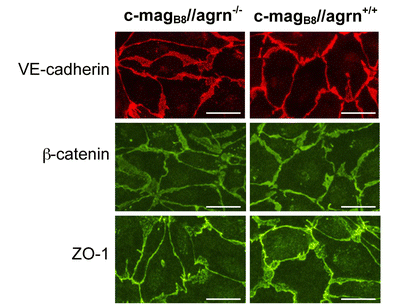

Supplement: Supplementary file 13 — Absence of agrin reduces junctional localization of VE-cadherin, β-catenin, and ZO-1 as demonstrated in Agrin−/− pMBMECs. Primary MBMECs isolated from rescued agrin knock-out (c-magB8//agrn−/−) or control littermates (c-magB8//agrn+/+) were stained for VE-cadherin, β-catenin, and ZO-1. Here, the junctional IF signal is displayed at high-power magnification for better visibility (for quantitative assessment, see Fig. 5). Micrographs are from one representative experiment out of 5. Bar 25 μm (GIF 59 kb) [file 441_2014_1969_Fig13_ESM.gif]

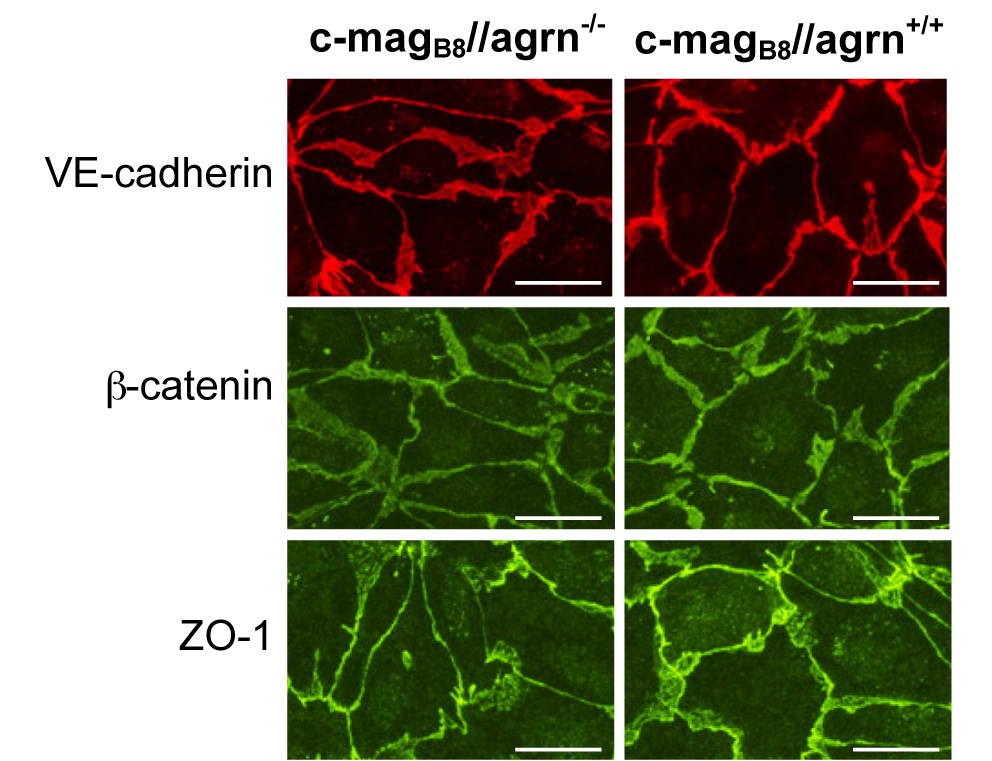

Supplement: Supplementary file 14 — High Resolution Image (TIFF 807 kb) [file 441_2014_1969_MOESM7_ESM.tif]
